# Rare Moss-Built Microterraces in a High-Altitude, Acid Mine Drainage-Polluted Stream (Cordillera Negra, Peru)

**DOI:** 10.1007/s11270-015-2390-x

**Published:** 2015-05-29

**Authors:** Jan Sevink, Jacobus M. Verstraten, Annemieke M. Kooijman, Raul A. Loayza-Muro, Leo Hoitinga, Edwin J. Palomino, Boris Jansen

**Affiliations:** Institute for Biodiversity and Ecosystem Dynamics, Amsterdam, The Netherlands; Laboratory of Ecotoxicology, Universidad Peruana Cayetano Heredia, Lima 31, Peru; Faculty of Environmental Sciences, Universidad Nacional ‘Santiago Antúnez de 31 Mayolo’, Huaraz, Peru

**Keywords:** Acid mine drainage, Arsenic, Bryophyte, Microterraces, Schwertmannite

## Abstract

The Rio Santiago in the Cordillera Negra of Peru is severely contaminated by acid mine drainage in its headwaters. In a strongly acid stream, at about 3800 m above sea level (masl), microterraces were found with terrace walls built up of dead moss, with encrustations and interstitial fine, creamy sediment. The stream water was turbid due to the presence of similar suspended sediment, which also occurred as a thin basal layer in inter-rim basins. The moss was identified as the rare bryophyte *Anomobryum prostratum* (Müll. Hal.) Besch. Chemical and mineralogical analyses show that green, living parts of the moss are gradually coated by Al/Fe (hydr)oxides, inducing their senescence and death. The necromass is covered by creamy crusts through precipitation of schwertmannite-type material from the stream water and simultaneous ‘capture’ of fine sediment. The latter consists of a mixture of precipitate and fine detrital primary minerals. These processes are held responsible for the formation of the microterraces, which regarding their composition and environment seem to be unique. Remarkable is the high As content of the creamy crusts and sediment, attributed to strong sorption of As, whereas its solute concentration is relatively low. This calls for more attention to suspended fine sediment in the assessment of environmental risks of stream water use. Lastly, the results raise serious doubts about the use of aquatic bryophytes as bioindicator for chemical pollution in acid mine drainage-polluted streams.

**Electronic supplementary material:**

The online version of this article (doi:10.1007/s11270-015-2390-x) contains supplementary material, which is available to authorized users.

## Introduction

**Fig. 1:**
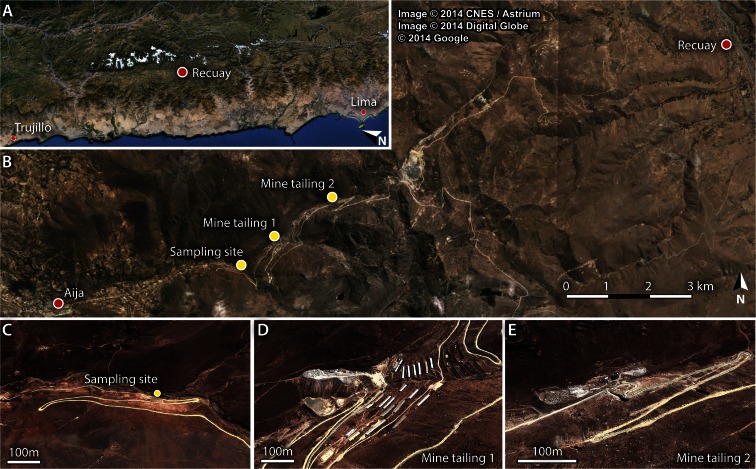
Location of the area of study (**a**, **b**), sampling site (**b**, **c**) and major upstream mine tailings (**d**, **e**)

The Cordillera Negra in Ancash (Peru) is noted for its polymetallic mines (Walsh 2013). Loayza-Muro et al. (2010) studied the heavy metal pollution and other environmental stress factors for the aquatic entomofauna in its high-altitude streams, inclusive of the Rio Santiago in the Aija catchment (see Fig. 1). During a visit to their Rio Santiago sampling site in November 2010 (end of the dry season), we observed hitherto unnoticed travertine-like microterraces that were built up by a single moss species; the only macro plant species found. Inter-rim basins held cream-coloured fine sediment, also encountered as interstitial fill in the moss rims.

Moss-built terraces are known from highly calcareous environments in which travertine is formed (e.g. Pentacost 2005), but not from such fundamentally different and extreme environments as that of the Rio Santiago: extremely acid, highly polluted, and at about 3800 m above sea level (masl) (see Table 1). This paper concerns a case study of such unusual aquatic system with emphasis on the composition and origin of the moss-built microterraces and fine suspended sediment, the identification of the moss species, and its survival in this extreme environment.

**Table 1:** Chemical composition of the stream water

	This study	Loayza et al.
	With filtration	Without filtration^b^
O_2_ (mg L^−1^)	nd	5
Temperature (°C)	nd	11.5
pH^a^	3.3	3.4
EC25 (microS cm^−1^)^a^	1960	1776
	μmol L^−1^	μmol L^−1^
K	114	34
Na	573	256
Ca	4110	1491
Mg	943	704
Sr	11.2	nd
Al	2.6	484
Fe	5000	671
Mn	1055	358
Zn	1030	246
Pb	<0.25	4.2
Cd	0.4	4.5
Si	341	445
SO_4_	12,210	nd
Cl	252	nd
As	104	47
ortho-P	1.9	1.8
NH_4_	415	1.8
NO_3_	<3	nd

Data from Loyaza et al. (2010) for the same stream with *n* = 4

*nd* not determined

^a^Comparable data

^b^Acidified with 10 M HNO_3_

## Environmental Setting

The catchment is in the central part of the Cordillera Negra, west of Recuay (Fig. 1). Data on its climate are truly scarce. It is a relatively dry tropical puna climate with distinct seasonal precipitation, which probably ranges from 500 to 1000 mm annually. Loayza-Muro et al. (2010) observed a roughly twofold increase in wet season discharge of the Rio Santiago at 3800 masl, while the water temperature ranged from 11.5 °C (wet season: December–April) to 9.3 °C (dry season), suggesting a similar small range in mean monthly air temperatures.

The main geological unit is the Early Tertiary Calipuy Group, composed of varied volcanic strata, but in places strata are non-volcanic and even some limestone beds were observed (Bodenlos and Straczek 1957). To the west, towards Aija, a large intrusive complex occurs, while some minor intrusive bodies have been found with associated polymetallic ore deposits close to the sampling site (MRC1 n.d; Chirif et al. 2010). Ores from several mines in the upper Rio Santiago catchment near the Huancapeti pass are processed in nearby plants, and residues are dumped in huge tailings and reservoirs (see Fig. 1). Waters from these dumps are very acid, pH values of 3 or even lower being reported by Loayza-Muro et al. (2010) and Walsh (2013).

Studies on the flora of the Cordillera Negra are rare and seemingly inexistent for the high, central part of this range. The few studies concern specific plants, such as *Brassica* spp. (Monsalve and Cano 2003) and lichens (Ramírez and Cano 2005). Reports on mosses are limited to a few early publications with descriptions of locations at which specific species were found (Zander and Hegewald 1976; Hegewald and Hegewald 1977 and 1985).

**Fig. 2:**
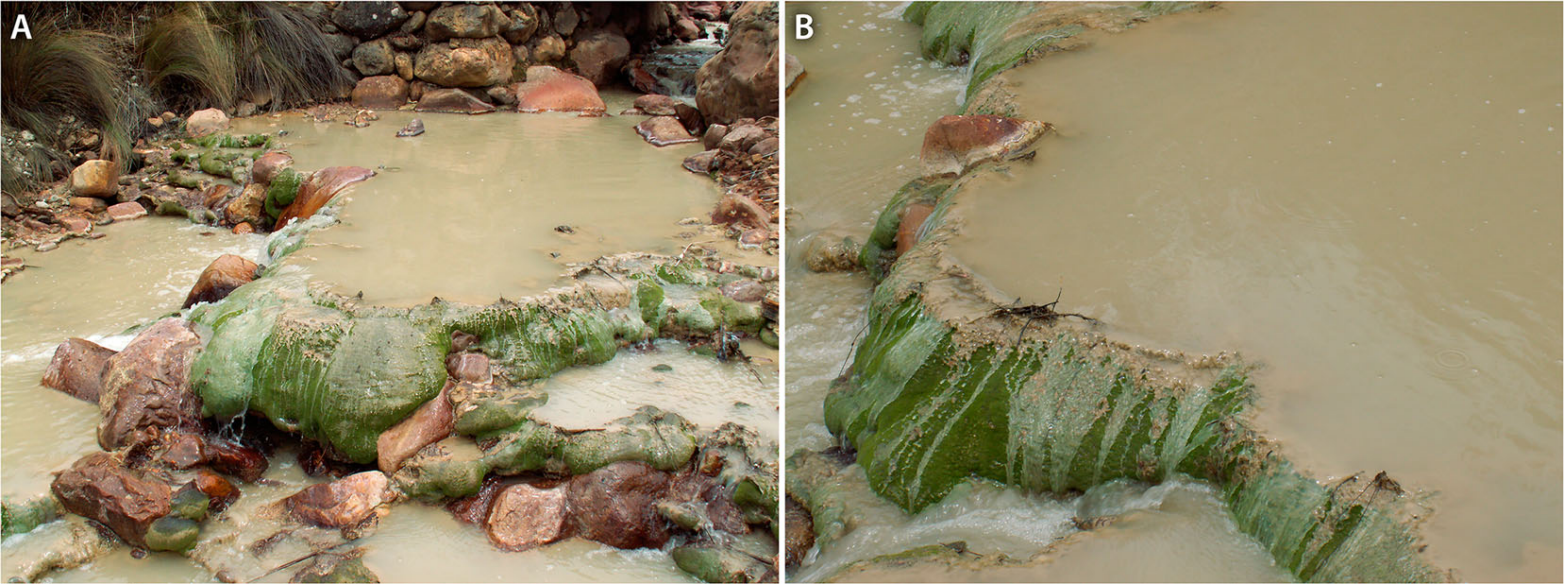
**a** The stream with its moss-built terraces and rust-stained coarse debris. **b** Detail of rim and basin showing the creamy sediment and hydrophobic moss surface

Figure 2 shows several aspects of the area around the sampling site, which is at 3800 masl. The riverbed is in the country rock (probably acid igneous rock; Chirif et al. 2010) and partially filled with very coarse textured and poorly sorted fluvial deposits. Iron hydroxides thinly coat the bedrock and sediment to the level reached during flood. The top of the microterraces built up by the moss is virtually horizontal. Rims are up to 2 m long and several decimetres high. Moss fills gaps in between the boulders and blocks, retaining water in small basins that may be several decimetres deep.

The stream is turbid, with very fine cream-coloured suspended material. In the basins, it has accumulated in a bottom layer that is up to several centimetres thick. Particles, when carried with the water over the rims, are partially caught by the moss (Fig. 2) and fill its interstices. General data on the stream are provided by Loayza-Muro et al. (2010): discharge during the dry season is about 50 L/s, mean temperature is about 10 °C (*n* = 4), and the oxygen content of the turbulent and thus oxygenated stream is about 5 mg/L (±0.74; *n* = 4).

No indications (e.g. high flood lines in the river bed or physical damage to the terrace rims) were found for exceptional rainstorms or dam breaks that might have led to incidental flushes of polluted water from the mine dumps upstream of the site in the previous period (the dry season), compromising the relevance of our water sampling for the longer-term stream water composition during this dry season, nor were such events reported by interviewed locals.

## Materials and Methods

Samples were taken on the 28th of November 2010, at the end of the dry season. Stream water and creamy material was sampled in small polyethylene bottles (*n* = 2). Bottles were stored at 4 °C in Peru and the Netherlands, interrupted by their transport (packed in insulating foam) to the Netherlands. They were filtered over a 0.2-μm membrane on the 2nd of December 2010 (e.g. 5 days later). Filtrates were combined into one water sample that was used for chemical analysis of solutes. After filtration, part of the water sample was acidified with HNO_3_, the other part remained untreated. Both were analysed within a month. The remaining material was combined into one sediment sample (sample P1), washed and centrifuged, subsequently freeze-dried and used for further analyses. Creamy sediment was resampled in November 2011 and after immediate transport to the Netherlands, washed and centrifuged, freeze-dried and also used for chemical analysis (sample P2).

To check for changes in stream water composition during transport and storage, pH and electrical conductivity (EC) values were established immediately after sampling in Peru and again prior to filtration in the lab (within 5 days after sampling). No changes in pH, EC, and colour of the creamy material were observed, as could be expected considering the short period of storage under appropriate conditions and the turbulent and oxygenated conditions in the stream.

**Fig. 3:**
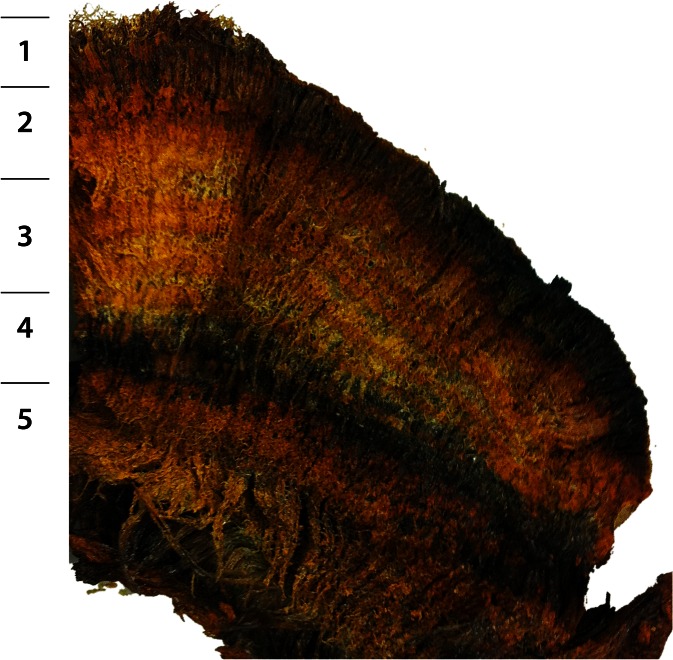
Cross-section with zones sampled (*1* = P3, *2* = P4, *3* = P5, *4* = P6, *5* = P7). Vertical section is about 10 cm

Moss samples were taken from one of the rims, packed in polyethylene bags and insulating foam and kept in a refrigerator at 4 °C in Peru and the Netherlands. Part was freeze-dried after washing to conserve the plant structure and used for its identification. The vertical stratification in the moss rims was studied by cutting slices from a large block as indicated in Fig. 3, washing these slices with demi-water over a sieve, followed by centrifugation and freeze-drying of the sediment obtained (P3–P7), and their chemical analysis. Chemical analyses were performed on samples of green, living moss carefully razor cut from the upper 3–4 mm of a moss monolith (see Figs. 2 and 3) to establish the composition of this plant material.

The creamy material (both suspended and moss-captured) was microscopically studied, using a Leitz petrographic microscope (magnification up to ×500), and both moss and creamy material were studied with a Leitz stereomicroscope at lower magnifications (up to ×50).

Chemical analyses were performed on moss and sediment samples using several methods:

- Method 1—after ignition of the dry sample (*n* = 2) to destroy organic matter and destruction in a hot HF/H_2_SO_4_ mixture, followed by dissolution of the remaining salts in hydrochloric acid (Jackson 1956), elements were estimated using a Perkin Elmer Optima 3000 XL ICP-OES (Perkin Elmer, MA, USA).

- Method 2—samples (*n* = 1) were dried, ignited to destroy organic matter and fused with lithium tetraborate into a bead (Van Reeuwijk 2002). Element concentrations were estimated by X-ray fluorescence spectroscopy (XRF).
- Method 3—total carbon (C), total nitrogen (N) and total sulphur (S) were estimated (*n* = 2) with an Elementar VarioEL elemental analyser (Elementar, Hanau, Germany) according to Van Reeuwijk (2002).
- Method 4—Fe(II) was established by destruction (*n* = 2) in a cold HF/H_2_SO_4_ solution and determined by colorimetric detection after complexation with 1.10-phenantroline (Van Reeuwijk 2002).
- Method 5—acid ammonium oxalate (AAO) extractable elements at pH 3.0 in the dark were determined (*n* = 2) as described by Van Reeuwijk (2002). In this extraction, all the amorphous material and schwertmannite plus ferrihydrite are dissolved.

Loss on ignition (LOI) was determined (*n* = 2) at 950 °C. X-ray diffraction (XRD) analysis was performed on oriented samples, prepared using a filter-membrane technique. Samples were scanned with a X-ray diffractometer with Cu Kα radiation. The acidified water sample was analysed in duplicate by inductively coupled plasma (ICP) (Perkin Elmer 3000XL OES, MA, USA) and the untreated water sample (single) by Continuous Flow Analyzer SAN++ (Skalar, Breda, the Netherlands). pH was estimated with a WTW pH meter (Weilheim, Germany) and EC_25_ with a WTW conductometer (Weilheim, Germany) with temperature compensation. The VISUAL MINTEQ version 3.0 program (KTH, Stockholm, Sweden) was used to calculate dissolved metal, sulphate and arsenate speciation and to estimate saturation indices for relevant minerals (e.g. Erten-Unal et al. 1998).

## Results

### Biotic Composition of the Terrace Rims

During the field sampling in November 2010, we found the terrace rims to be composed of a single moss species. Microscopic study of the terrace rim sample confirmed our field observation on the plant species composition, only one moss species being present. Since the moss had no fructifications, we repeatedly revisited the site over the next 2 years to see whether fructifications were present and eventually collect these for identification, but they were not encountered.

**Fig. 4:**
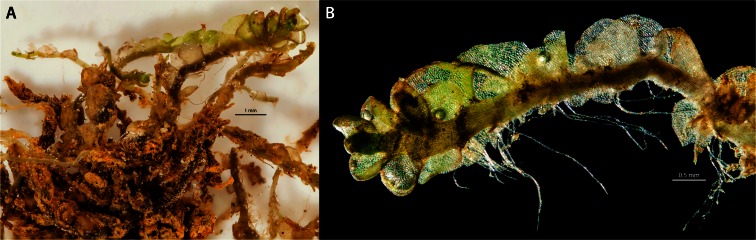
Microphotographs of the moss showing the development of the coatings: green living moss, thin translucent coatings on still living tissue (**b**, *right side*) and thick coatings on dead plant material (**a**)

The identification of the moss as *Anomobryum prostratum* (Müll. Hal.) Besch, by William R. Buck (New York Botanical Garden), was based on morphological characteristics. Confirmation of this identification by molecular genetic data was impossible, since these do not yet exist for the genus (see, e.g. GenBank: www.ncbi.nlm.nih.gov/genbank/). The sample identified has been deposited in the herbarium of the New York Botanical Garden. The morphological characteristics of the species (see Figs. 2, 3 and 4) and its distribution are described in considerable detail in the Tropicos archive (Tropicos.org) and in other publications on this species (e.g. Hegewald and Hegewald et al. 1977 and 1985).

*A. prostratum* is a rare South American species from the Bryaceae family that occurs from Mexico to Argentina, usually at higher altitudes, i.e. between 1800 and 4285 masl (GBIF 2013). This species belongs to a worldwide *Anomobryum* genus of 50–60 species, which are most common in montane regions in the southern hemisphere, especially in the Neotropics, and generally occur on damp soil and rock (Spence and Ramsey 2002). In the Tropicos archive (Tropicos.org), the habitat of *A. prostratum* is described as ‘on wet soil, bare road-cut soil, moist walls, and boulders in forest or along streams’, but the species also occurs in hot springs (GBIF 2013). Hegewald and Hegewald (1977, 1985) found *A. prostratum* in 1973 in the nearby Catac area. One of the occurrences was at 4100 masl, and Hegewald and Hegewald (Tropicos.org) reported even higher altitudes for Peru.

**Fig. 5:**
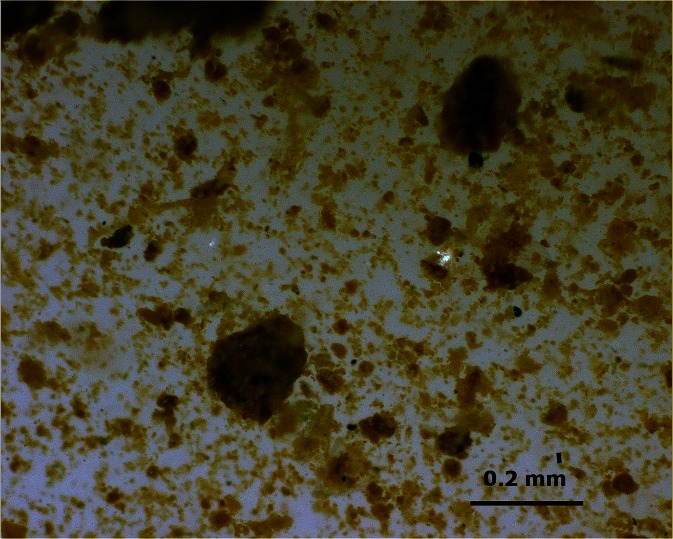
Microphotograph of suspended material from the stream

Under the microscope, bacterial and algal colonies in the form of films, slimes or other types of colonies were not observed, neither on the surface of the living moss (which is hydrophobic) nor inside the moss-built terrace rim sampled (see also Section 4.2). Neither were microbial structures (bacterial or algal colonies) visible at the magnifications used (up to ×500) in the suspended sediment present in the stream water and in the interstitial sediment of the moss rim sampled (see Fig. 5).

### Terrace Rim Structure and Chemical Composition

A cross-section of a rim is shown in Fig. 3 evidencing the internal structure. Only the outer part (less than 1 cm) consisted of green plant parts, and these were hydrophobic, as can be seen in Fig. 2. The remainder consisted of a dense fibrous mass of (macroscopically) moderately encrusted dead moss fibres (necromass) and, in between the fibres, loose creamy to brownish sediment. Remarkable was the occurrence of alternating lighter and more brownish, darker coloured bands, resulting from slight differences in the colour of the moss encrustations. Thicknesses of individual successions (lighter and darker layers) were of the same order as the thickness of the living moss layer, suggesting a seasonal rhythm (dry-wet season) in the built-up of the moss rim.

Figure 4 illustrates the main characteristics of the moss at microscopic scale. The green plant material is largely covered by a thin translucent coating of creamy to brownish, optically isotic material. Going from the green youngest parts downward, the coating changes from this translucent coating to a relatively dense coating composed of very fine-grained creamy material cementing fine crystalline mineral fragments, with dead plant material inside.

Chemical analyses of green, living moss were evidently hampered by the coatings, which appeared to be hard to remove. A moss sample washed with water had a C content of approx. 24 %, acid-washed moss 33 % and samples that were thoroughly pretreated (ultrasonic treatment and washing with demi-water) had slightly over 40 % and a weight loss on ignition of near 95 %. Element concentrations of these various moss samples are given in Table 2. Microscopic study revealed that in the thoroughly pretreated samples, the living moss still had some thin translucent coatings, while dense creamy coatings were absent, whereas in the other samples such relatively dense coatings were common, explaining the observed differences in composition.

**Table 2:** Chemical composition of the moss after different pretreatments (chemical elements in mmol kg^−1^ and oxides in weight %, both on absolute dry base)

	Moss water		Moss acid		Moss 1 ultrasonic		Moss 2 ultrasonic	
	mmol kg^−1^	%	mmol kg^−1^	%	mmol kg^−1^	%	mmol kg^−1^	%
K/K_2_O	582	2.74	566	2.67	389	1.83	244	1.15
Na/Na_2_O	230	0.71	208	0.64	92.4	0.29	52.3	0.16
Ca/CaO	98.2	0.55	62.6	0.35	136	0.76	83.7	0.47
Mg/MgO	170	0.69	144	0.58	73.6	0.30	61.5	0.25
Al/Al_2_O_3_	950	4.84	642	3.27	183	0.93	128	0.65
Fe/Fe_2_O_3_	605	4.83	459	3.67	153	1.22	138	1.10
Mn/MnO	7.46	<<0.1	4.18	<<0.1	1.1	<<0.1	0.58	<<0.1
Zn/ZnO	16.5	<<0.1	11	<<0.1	8.9	<<0.1	6.67	<<0.1
Pb/PbO	24	<<0.1	14	<<0.1	3.8	<<0.1	2.47	<<0.1
As/As_2_O_5_	88.3	1.01	81.4	0.94	10.4	0.12	7.39	0.08
P/P_2_O_5_	40.4	0.38	45	0.43	46.1	0.44	27.9	0.26
S/SO_3_^a^	430	2.20	307	1.57	bdl	bdl	bdl	bdl
LOI		60.9		70.4		94.0		95.6
C^a^		28.7		33.0		40.7		41.2

*bdl* below detection limit

^a^Based on CNS analyser

### Water Chemistry

The data on the water sample taken in 2010 demonstrate the highly acidic nature of the stream water (Table 1). Dissolved Fe is present in large amounts, as are Ca and SO_4_. As to the occurrence of heavy metals and related elements, notable is the concentration of As (104 μmol L^−1^) and the low concentrations of heavy metals with the exception of Zn. Differences in composition relative to the results of Loayza et al. (2010) are evident and primarily concern element concentrations, whereas pH and EC_25_ values are similar (indicated with a superscript ‘a’ in Table 1). Given the latter, the considerably lower concentration of most elements in the samples of Loayza et al. (2010) is surprising. Unfortunately, their results cannot be checked for their reliability on the basis of the electroneutrality principle, since S was not estimated. It should be emphasized that in the turbulent stream, Loayza et al. (2010) found mean oxygen concentrations of 5 mg/L, which is close to oxygen saturation for this temperature and altitude (Jacobsen 2011) and testifies to the aerobic conditions in the stream.

### Sediment Composition

Elemental analyses are presented in Table 3. Fe contents are expressed as Fe_2_O_3_, S as SO_3_ and As as As_2_O_5_. Si contents could not be established by method 1 and therefore were calculated (Si = a * b) from (a) measured Si/Al ratios found by XRF (method 2, only performed on samples P1 and P2) and (b) values found for Al by the ICP analyses (method 1). Fe(II) contents of the samples P1–7 (method 4) were in the order of 3–5 % of the total Fe, expressed as oxides, evidencing that virtually all Fe occurs as Fe(III). In principle, S might be present as sulphide, taking into account the nature of the sediment (derived from tailings of sulphidic ores). However, given the prominent aerated nature of the stream water (see, e.g. dissolved oxygen contents in Table 1) and the low concentrations of heavy metals, it is unlikely that more than traces of sulphides are present. Moreover, in the X-ray analyses (samples P1 and P2), no indication was found for the presence of Fe sulphide or other heavy metal sulphides in detectable concentrations. Arsenic is present as As_2_O_5_, since this As occurs as dissolved species in an oxidative environment (Tables 4, 5 and 6).

**Table 3:** Chemical composition of the sediment samples (weight % or ppm on absolute dry base) based on methods 1 (total) and 5 (AAO: amorph), unless indicated otherwise

	P1		P2		P3	P4	P5	P6	P7
	Total	Amorph	Total	Amorph	Total	Total	Total	Total	Total
Weight %									
SiO_2_^a^	22.6	0.07	26.5	0.10	nd	nd	nd	nd	nd
K_2_O	1.63	0.04	1.69	0.01	0.51	1.69	1.61	1.04	1.50
Na_2_O	0.52	<0.01	0.51	<0.01	0.10	0.49	0.53	0.21	0.49
CaO	0.71	0.01	0.68	0.01	0.38	0.82	0.96	0.48	0.75
MgO	0.84	0.02	0.87	0.01	0.29	0.82	0.81	0.47	0.68
Al_2_O_3_	8.58	0.11	9.29	0.25	2.62	8.26	8.51	5.39	7.98
Fe_2_O_3_	33.2	23.4	29.2	21.1	45.6	30.0	27.6	32.8	31.1
TiO_2_	0.40	<0.01	0.41	<0.01	nd	nd	nd	nd	nd
P_2_O_5_	0.35	0.22	0.39	0.28	<0.01	0.35	0.36	bdl	0.47
SO_3_^b^	6.93	3.44^c^	5.55	2.68^c^	6.61	6.53	7.44	8.01	6.23
FeO by method 4	0.91		1.12		2.28	1.17	1.09	1.27	0.83
Content in ppm									
Mn	438	22	657	107	785	653	645	664	575
Zn	1910	97	2120	346	2980	1630	1280	1350	1330
Pb	4450	nd	5400	nd	1340	3440	5350	3530	5500
Cu	150	20	159	31	79	204	321	651	369
Sr	81	nd	91	nd	34	97	106	47	91
As	21,400	15,700	24,800	17,900	12,300	17,800	23,600	20,200	36,500
Weight %									
C^b^	1.99		1.95		12.17	2.75	1.86	11.95	3.22
N^b^	0.20		0.16		0.72	0.23	0.15	0.49	0.18
S^b^	2.77		2.22		3.13	2.51	3.10	2.83	2.32
LOI	15.50		12.52		28.63	10.24	7.50	29.35	10.42

Values for elements are expressed in at most three significant digits

*nd* not determined

^a^Calculated from X-ray fluorescence

^b^CNS analyser

^c^By ICP

**Table 4:** Log IAP, sat. index and stoichiometry of minerals

Mineral	Log IAP	Sat. index	Stoichiometry									
Al(OH)_3_ (soil)	2.9	−6.262	1	Al^+3^	3	H_2_O	−3	H^+1^				
Al_2_O_3_(s)	5.8	−16.001	2	Al^+3^	3	H_2_O	−6	H^+1^				
Al_4_(OH)_10_SO_4_(s)	2.496	−20.204	−10	H^+1^	4	Al^+3^	1	SO_4_^−2^	10	H_2_O		
AlAsO_4_:_2_H_2_O(s)	−23.45	−7.65	1	Al^+3^	1	AsO_4_^−3^	2	H_2_O				
AlOHSO_4_(s)	−6.204	−2.974	−1	H^+1^	1	Al^+3^	1	SO_4_^−2^	1	H_2_O		
Alunite	−10.228	−10.573	1	K^+1^	3	Al^+3^	2	SO_4_^−2^	−6	H^+1^	6	H_2_O
Anglesite	−9.649	−1.759	1	Pb^+2^	1	SO_4_^−2^						
Anhydrite	−5.293	−0.993	1	Ca^+2^	1	SO_4_^−2^						
Antlerite	−13.6	−22.388	3	Cu^+2^	4	H_2_O	−4	H^+1^	1	SO_4_^−2^		
Aragonite	−29.105	−20.843	1	Ca^+2^	1	CO_3_^−2^						
Artinite	−26.532	−37.131	−2	H^+1^	2	Mg^+2^	1	CO_3_^−2^	5	H_2_O		
As_2_O_5_(s)	−52.7	−17.983	2	AsO_4_^−3^	6	H^+1^	−3	H_2_O				
Atacamite	−9.97	−18.137	2	Cu^+2^	3	H_2_O	−3	H^+1^	1	Cl^−1^		
Azurite	−70.328	−53.265	3	Cu^+2^	2	H_2_O	−2	H^+1^	2	CO_3_^−2^		
Bianchite	−5.898	−4.138	1	Zn^+2^	1	SO_4_^−2^	6	H_2_O				
Boehmite	2.9	−6.656	−3	H^+1^	1	Al^+3^	2	H_2_O				
Brochantite	−15.098	−32.006	4	Cu^+2^	6	H_2_O	−6	H^+1^	1	SO_4_^−2^		
Brucite	3.192	−14.855	1	Mg^+2^	2	H_2_O	−2	H^+1^				
Ca_3_(AsO_4_)_2_:_4_H_2_O(s)	−41.267	−22.367	3	Ca^+2^	2	AsO_4_^−3^	4	H_2_O				
Ca_3_(PO_4_)_2_ (am_1_)	−49.986	−25.267	3	Ca^+2^	2	PO_4_^−3^	−3	H^+1^				
Ca_3_(PO_4_)_2_ (am_2_)	−49.986	−22.459	3	Ca^+2^	2	PO_4_^−3^	−3	H^+1^				
Ca_3_(PO_4_)_2_ (beta)	−49.986	−20.617	3	Ca^+2^	2	PO_4_^−3^						
Ca_4_H(PO_4_)_3_:_3_H_2_O(s)	−76.884	−29.806	4	Ca^+2^	1	H^+1^	3	PO_4_^−3^	3	H_2_O		
CaCO_3_xH_2_O(s)	−29.105	−22.03	1	Ca^+2^	1	CO_3_^−2^	1	H_2_O				
CaHPO_4_(s)	−26.898	−7.366	1	Ca^+2^	1	H^+1^	1	PO_4_^−3^				
CaHPO_4_:_2_H_2_O(s)	−26.898	−7.712	1	Ca^+2^	1	H^+1^	1	PO_4_^−3^	2	H_2_O		
Calcite	−29.105	−20.689	1	Ca^+2^	1	CO_3_^−2^						
Cd(OH)_2_(s)	−0.214	−14.644	1	Cd^+2^	2	H_2_O	−2	H^+1^				
Cd_3_(OH)_4_SO_4_(s)	−9.745	−32.305	−4	H^+1^	3	Cd^+2^	4	H_2_O	1	SO_4_^−2^		
Cd_3_(PO_4_)_2_(s)	−62.059	−29.459	3	Cd^+2^	2	PO_4_^−3^						
Cd_3_OH_2_(SO_4_)_2_(s)	−18.849	−25.559	−2	H^+1^	3	Cd^+2^	2	H_2_O	2	SO_4_^−2^		
Cd_4_(OH)_6_SO_4_(s)	−9.959	−38.359	−6	H^+1^	4	Cd^+2^	6	H_2_O	1	SO_4_^−2^		
CdCl_2_(s)	−14.159	−13.654	1	Cd^+2^	2	Cl^−1^						
CdCl_2_:_1_H_2_O(s)	−14.159	−12.528	1	Cd^+2^	2	Cl^−1^	1	H_2_O				
CdCl_2_:_2_._5_H_2_O(s)	−14.159	−12.185	1	Cd^+2^	2	Cl^−1^	2.5	H_2_O				
CdOHCl(s)	−7.186	−10.98	−1	H^+1^	1	Cd^+2^	1	H_2_O	1	Cl^−1^		
CdSO_4_(s)	−9.318	−9.577	1	Cd^+2^	1	SO_4_^−2^						
CdSO_4_:_1_H_2_O(s)	−9.318	−7.854	1	Cd^+2^	1	SO_4_^−2^	1	H_2_O				
CdSO_4_:_2_._67_H_2_O(s)	−9.318	−7.594	1	Cd^+2^	1	SO_4_^−2^	2.67	H_2_O				
Celestite	−7.842	−1.205	1	Sr^+2^	1	SO_4_^−2^						
Cerussite	−33.461	−20.055	1	Pb^+2^	1	CO_3_^−2^						
Chalcanthite	−10.603	−7.913	1	Cu^+2^	1	SO_4_^−2^	5	H_2_O				
Chalcedony	−3.465	0.249	1	H_4_SiO_4_	−2	H_2_O						
Chloropyromorphite(c)	−101.825	−17.395	5	Pb^+2^	3	PO_4_^−3^	1	Cl^−1^				
Chloropyromorphite(soil)	−101.825	−21.425	5	Pb^+2^	3	PO_4_^−3^	1	Cl^−1^				
Chrysotile	2.646	−31.182	3	Mg^+2^	2	H_4_SiO_4_	1	H_2_O	−6	H^+1^		
Cotunnite	−14.49	−9.493	1	Pb^+2^	2	Cl^−1^						
Cristobalite	−3.465	0.051	1	H_4_SiO_4_	−2	H_2_O						
Cu(OH)_2_(s)	−1.499	−11.23	1	Cu^+2^	2	H_2_O	−2	H^+1^				
Cu_2_(OH)_3_NO_3_(s)	−12.893	−22.747	2	Cu^+2^	3	H_2_O	−3	H^+1^	1	NO_3_^−1^		
Cu_3_(AsO_4_)_2_:_2_H_2_O(s)	−57.195	−22.095	3	Cu^+2^	2	AsO_4_^−3^	2	H_2_O				
Cu_3_(PO_4_)_2_(s)	−65.914	−29.064	3	Cu^+2^	2	PO_4_^−3^						
Cu_3_(PO_4_)_2_:_3_H_2_O(s)	−65.914	−30.794	3	Cu^+2^	2	PO_4_^−3^	3	H_2_O				
CuCO_3_(s)	−34.415	−22.915	1	Cu^+2^	1	CO_3_^−2^						
CuOCuSO_4_(s)	−12.101	−23.549	−2	H^+1^	2	Cu^+2^	1	H_2_O	1	SO_4_^−2^		
Cupric ferrite	9.832	2.097	−8	H^+1^	1	Cu^+2^	2	Fe^+3^	4	H_2_O		
CuSO_4_(s)	−10.603	−14.149	1	Cu^+2^	1	SO_4_^−2^						
Diaspore	2.9	−4.829	−3	H^+1^	1	Al^+3^	2	H_2_O				
Dolomite (disordered)	−58.829	−42.675	1	Ca^+2^	1	Mg^+2^	2	CO_3_^−2^				
Dolomite (ordered)	−58.829	−42.067	1	Ca^+2^	1	Mg^+2^	2	CO_3_^−2^				
Epsomite	−5.912	−3.689	1	Mg^+2^	1	SO_4_^−2^	7	H_2_O				
Ettringite	1.353	−58.693	6	Ca^+2^	2	Al^+3^	3	SO_4_^−2^	−12	H^+1^	38	H_2_O
Fe(OH)_2_ (am)	2.891	−11.361	1	Fe^+2^	2	H_2_O	−2	H^+1^				
Fe(OH)_2_ (c)	2.891	−9.999	1	Fe^+2^	−2	H^+1^	2	H_2_O				
Fe(OH)_2_._7_Cl._3_(s)	3.574	6.614	−2.7	H^+1^	1	Fe^+3^	2.7	H_2_O	0.3	Cl^−1^		
Fe_2_(SO_4_)_3_(s)	−15.981	−14.258	2	Fe^+3^	3	SO_4_^−2^						
Fe_3_(OH)_8_(s)	14.221	−6.001	−8	H^+1^	2	Fe^+3^	1	Fe^+2^	8	H_2_O		
FeAsO_4_:_2_H_2_O(s)	−20.684	−0.484	1	Fe^+3^	1	AsO_4_^−3^	2	H_2_O				
Ferrihydrite	5.665	1.631	1	Fe^+3^	3	H_2_O	−3	H^+1^				
Ferrihydrite (aged)	5.665	2.141	1	Fe^+3^	−3	H^+1^	3	H_2_O				
Gibbsite (C)	2.9	−5.712	1	Al^+3^	3	H_2_O	−3	H^+1^				
Goethite	5.665	4.671	1	Fe^+3^	2	H_2_O	−3	H^+1^				
Goslarite	−5.898	−3.769	1	Zn^+2^	1	SO_4_^−2^	7	H_2_O				
Greenalite	1.742	−19.068	−6	H^+1^	3	Fe^+2^	2	H_4_SiO_4_	1	H_2_O		
Gypsum	−5.293	−0.675	1	Ca^+2^	1	SO_4_^−2^	2	H_2_O				
Halite	−6.992	−8.511	1	Na^+1^	1	Cl^−1^						
Halloysite	−1.13	−12.212	2	Al^+3^	2	H_4_SiO_4_	1	H_2_O	−6	H^+1^		
Hematite	11.331	11.677	2	Fe^+3^	3	H_2_O	−6	H^+1^				
Hercynite	8.691	−16.81	−8	H^+1^	1	Fe^+2^	2	Al^+3^	4	H_2_O		
Hinsdalite	−31.658	−29.158	−6	H^+1^	1	Pb^+2^	3	Al^+3^	1	PO_4_^−3^	1	SO_4_^−2^
H-Jarosite	−1.212	2.323	3	Fe^+3^	2	SO_4_^−2^	−5	H^+1^	7	H_2_O		
Huntite	−118.277	−89.205	3	Mg^+2^	1	Ca^+2^	4	CO_3_^−2^				
Hydrocerrusite	−67.467	−48.707	3	Pb^+2^	2	H_2_O	−2	H^+1^	2	CO_3_^−2^		
Hydromagnesite	−115.704	−108.753	5	Mg^+2^	4	CO_3_^−2^	−2	H^+1^	6	H_2_O		
Hydroxyapatite	−73.073	−28.74	5	Ca^+2^	3	PO_4_^−3^	1	H_2_O	−1	H^+1^		
Hydroxylpyromorphite	−94.852	−32.062	5	Pb^+2^	3	PO_4_^−3^	1	H_2_O	−1	H^+1^		
Hydrozincite	−49.801	−60.466	5	Zn^+2^	2	CO_3_^−2^	−6	H^+1^	6	H_2_O		
Imogolite	2.335	−12.273	2	Al^+3^	1	H_4_SiO_4_	3	H_2_O	−6	H^+1^		
K-Alum	−16.029	−10.608	1	K^+1^	1	Al^+3^	2	SO_4_^−2^	12	H_2_O		
Kaolinite	−1.13	−9.794	2	Al^+3^	2	H_4_SiO_4_	1	H_2_O	−6	H^+1^		
KCl(s)	−7.693	−8.593	1	K^+1^	1	Cl^−1^						
K-Jarosite	−1.932	8.222	1	K^+1^	3	Fe^+3^	2	SO_4_^−2^	−6	H^+1^	6	H_2_O
Langite	−15.098	−33.962	−6	H^+1^	4	Cu^+2^	7	H_2_O	1	SO_4_^−2^		
Larnakite	−10.194	−9.941	−2	H^+1^	2	Pb^+2^	1	SO_4_^−2^	1	H_2_O		
Laurionite	−7.518	−8.141	−1	H^+1^	1	Pb^+2^	1	Cl^−1^	1	H_2_O		
Lepidocrocite	5.665	4.294	−3	H^+1^	1	Fe^+3^	2	H_2_O				
Lime	3.811	−30.5	−2	H^+1^	1	Ca^+2^	1	H_2_O				
Litharge	−0.545	−13.779	1	Pb^+2^	1	H_2_O	−2	H^+1^				
Maghemite	11.331	4.945	−6	H^+1^	2	Fe^+3^	3	H_2_O				
Magnesioferrite	14.523	−4.654	−8	H^+1^	1	Mg^+2^	2	Fe^+3^	4	H_2_O		
Magnesite	−29.724	−22.098	1	Mg^+2^	1	CO_3_^−2^						
Magnetite	14.221	9.086	−8	H^+1^	2	Fe^+3^	1	Fe^+2^	4	H_2_O		
Malachite	−35.913	−30.811	2	Cu^+2^	2	H_2_O	−2	H^+1^	1	CO_3_^−2^		
Massicot	−0.545	−13.991	1	Pb^+2^	1	H_2_O	−2	H^+1^				
Melanothallite	−15.444	−22.228	1	Cu^+2^	2	Cl^−1^						
Melanterite	−6.213	−3.834	1	Fe^+2^	1	SO_4_^−2^	7	H_2_O				
Mg(OH)_2_ (active)	3.192	−15.602	1	Mg^+2^	2	H_2_O	−2	H^+1^				
Mg_2_(OH)_3_Cl:_4_H_2_O(s)	−0.588	−26.588	2	Mg^+2^	1	Cl^−1^	−3	H^+1^	7	H_2_O		
Mg_3_(PO_4_)_2_(s)	−51.842	−28.562	3	Mg^+2^	2	PO_4_^−3^						
MgCO_3_:_5_H_2_O(s)	−29.724	−25.184	1	Mg^+2^	1	CO_3_^−2^	5	H_2_O				
MgHPO_4_:_3_H_2_O(s)	−27.517	−9.342	1	Mg^+2^	1	H^+1^	1	PO_4_^−3^	3	H_2_O		
Mirabilite	−9.142	−7.368	2	Na^+1^	1	SO_4_^−2^	10	H_2_O				
Mn_3_(AsO_4_)_2_:_8_H_2_O(s)	−42.957	−14.257	3	Mn^+2^	2	AsO_4_^−3^	8	H_2_O				
Mn_3_(PO_4_)_2_(s)	−51.676	−27.775	3	Mn^+2^	2	PO_4_^−3^						
MnCl_2_:_4_H_2_O(s)	−10.698	−13.503	1	Mn^+2^	2	Cl^−1^	4	H_2_O				
MnCO_3_ (am)	−29.669	−19.169	1	Mn^+2^	1	CO_3_^−2^						
MnHPO_4_(s)	−27.462	−2.062	1	Mn^+2^	1	PO_4_^−3^	1	H^+1^				
MnSO_4_(s)	−5.857	−8.978	1	Mn^+2^	1	SO_4_^−2^						
Na-Jarosite	−1.231	2.961	1	Na^+1^	3	Fe^+3^	2	SO_4_^−2^	−6	H^+1^	6	H_2_O
Natron	−32.954	−31.096	2	Na^+1^	1	CO_3_^−2^	10	H_2_O				
Nesquehonite	−29.724	−25.255	1	Mg^+2^	1	CO_3_^−2^	3	H_2_O				
Otavite	−33.13	−21.063	1	Cd^+2^	1	CO_3_^−2^						
Pb(OH)_2_(s)	−0.545	−9.181	−2	H^+1^	1	Pb^+2^	2	H_2_O				
Pb_10_(OH)_6_O(CO_3_)_6_(s)	−202.947	−194.187	10	Pb^+2^	6	CO_3_^−2^	7	H_2_O	−8	H^+1^		
Pb_2_(OH)_3_Cl(s)	−8.063	−16.856	−3	H^+1^	2	Pb^+2^	3	H_2_O	1	Cl^−1^		
Pb_2_O(OH)_2_(s)	−1.09	−27.28	2	Pb^+2^	3	H_2_O	−4	H^+1^				
Pb_2_OCO_3_(s)	−34.006	−33.788	−2	H^+1^	2	Pb^+2^	1	H_2_O	1	CO_3_^−2^		
Pb_3_(AsO_4_)_2_(s)	−54.335	−18.835	3	Pb^+2^	2	AsO_4_^−3^						
Pb_3_(PO_4_)_2_(s)	−63.053	−19.523	3	Pb^+2^	2	PO_4_^−3^						
Pb_3_O_2_CO_3_(s)	−34.551	−46.49	−4	H^+1^	3	Pb^+2^	1	CO_3_^−2^	2	H_2_O		
Pb_3_O_2_SO_4_(s)	−10.739	−22.083	−4	H^+1^	3	Pb^+2^	1	SO_4_^−2^	2	H_2_O		
Pb_4_(OH)_6_SO_4_(s)	−11.285	−32.385	−6	H^+1^	4	Pb^+2^	1	SO_4_^−2^	6	H_2_O		
Pb_4_O_3_SO_4_(s)	−11.285	−34.295	−6	H^+1^	4	Pb^+2^	1	SO_4_^−2^	3	H_2_O		
PbHPO_4_(s)	−31.254	−7.449	1	Pb^+2^	1	H^+1^	1	PO_4_^−3^				
PbO:_0_._3_H_2_O(s)	−0.545	−13.525	−2	H^+1^	1	Pb^+2^	1.33	H_2_O				
Periclase	3.192	−19.649	−2	H^+1^	1	Mg^+2^	1	H_2_O				
Phosgenite	−47.951	−28.141	2	Pb^+2^	2	Cl^−1^	1	CO_3_^−2^				
Plumbgummite	−53.263	−20.473	−5	H^+1^	1	Pb^+2^	3	Al^+3^	2	PO_4_^−3^	6	H_2_O
Portlandite	3.811	−19.962	1	Ca^+2^	2	H_2_O	−2	H^+1^				
Pyrochroite	3.247	−12.753	1	Mn^+2^	2	H_2_O	−2	H^+1^				
Quartz	−3.465	0.721	1	H_4_SiO_4_	−2	H_2_O						
Rhodochrosite	−29.669	−18.684	1	Mn^+2^	1	CO_3_^−2^						
Sepiolite	−4.011	−20.719	2	Mg^+2^	3	H_4_SiO_4_	−4	H^+1^	−0.5	H_2_O		
Sepiolite (A)	−4.011	−22.791	−0.5	H_2_O	2	Mg^+2^	3	H_4_SiO_4_	−4	H^+1^		
Siderite	−30.025	−19.496	1	Fe^+2^	1	CO_3_^−2^						
SiO_2_ (am,gel)	−3.465	−0.639	1	H_4_SiO_4_	−2	H_2_O						
SiO_2_ (am,ppt)	−3.465	−0.599	1	H_4_SiO_4_	−2	H_2_O						
Smithsonite	−29.71	−18.841	1	Zn^+2^	1	CO_3_^−2^						
Spinel	8.992	−31.079	−8	H^+1^	1	Mg^+2^	2	Al^+3^	4	H_2_O		
SrHPO_4_(s)	−29.447	−10.152	1	Sr^+2^	1	H^+1^	1	PO_4_^−3^				
Strengite	−25.044	1.279	1	Fe^+3^	1	PO_4_^−3^	2	H_2_O				
Strontianite	−31.654	−22.392	1	Sr^+2^	1	CO_3_^−2^						
Tenorite(am)	−1.499	−10.528	1	Cu^+2^	1	H_2_O	−2	H^+1^				
Tenorite(c)	−1.499	−9.678	1	Cu^+2^	−2	H^+1^	1	H_2_O				
Thenardite	−9.142	−9.54	2	Na^+1^	1	SO_4_^−2^						
Thermonatrite	−32.954	−33.678	2	Na^+1^	1	CO_3_^−2^	1	H_2_O				
Tsumebite	−33.298	−23.508	−3	H^+1^	2	Pb^+2^	1	Cu^+2^	1	PO_4_^−3^	6	H_2_O
Vaterite	−29.105	−21.293	1	Ca^+2^	1	CO_3_^−2^						
Vivianite	−52.746	−14.944	3	Fe^+2^	2	PO_4_^−3^	8	H_2_O				
Zincite	3.206	−8.768	1	Zn^+2^	1	H_2_O	−2	H^+1^				
Zincosite	−5.898	−10.514	1	Zn^+2^	1	SO_4_^−2^						
Zn(NO_3_)_2_:_6_H_2_O(s)	−16.586	−19.697	1	Zn^+2^	2	NO_3_^−1^	6	H_2_O				
Zn(OH)_2_ (am)	3.206	−9.979	1	Zn^+2^	2	H_2_O	−2	H^+1^				
Zn(OH)_2_ (beta)	3.206	−9.239	1	Zn^+2^	2	H_2_O	−2	H^+1^				
Zn(OH)_2_ (delta)	3.206	−8.638	1	Zn^+2^	−2	H^+1^	2	H_2_O				
Zn(OH)_2_ (epsilon)	3.206	−8.981	1	Zn^+2^	2	H_2_O	−2	H^+1^				
Zn(OH)_2_ (gamma)	3.206	−9.208	1	Zn^+2^	2	H_2_O	−2	H^+1^				
Zn_2_(OH)_2_SO_4_(s)	−2.692	−10.192	−2	H^+1^	2	Zn^+2^	2	H_2_O	1	SO_4_^−2^		
Zn_2_(OH)_3_Cl(s)	−0.56	−15.751	2	Zn^+2^	3	H_2_O	−3	H^+1^	1	Cl^−1^		
Zn_3_(PO_4_)_2_:_4_H_2_O(s)	−51.8	−16.38	3	Zn^+2^	2	PO_4_^−3^	4	H_2_O				
Zn_3_AsO_42_:_2_._5_H_2_O(s)	−43.081	−15.581	3	Zn^+2^	2	AsO_4_^−3^	2.5	H_2_O				
Zn_3_O(SO_4_)_2_(s)	−8.59	−29.647	−2	H^+1^	3	Zn^+2^	2	SO_4_^−2^	1	H_2_O		
Zn_4_(OH)_6_SO_4_(s)	3.721	−24.679	−6	H^+1^	4	Zn^+2^	6	H_2_O	1	SO_4_^−2^		
Zn_5_(OH)_8_Cl_2_(s)	2.086	−36.414	−8	H^+1^	5	Zn^+2^	8	H_2_O	2	Cl^−1^		
Zn-Al LDH(s)	−7.146	−26.976	2	Zn^+2^	1	Al^+3^	0.5	CO_3_^−2^	−6	H^+1^	6	H_2_O
ZnCl_2_(s)	−10.739	−18.391	1	Zn^+2^	2	Cl^−1^						
ZnCO_3_(s)	−29.71	−18.91	1	Zn^+2^	1	CO_3_^−2^						
ZnCO_3_:_1_H_2_O(s)	−29.71	−19.45	1	Zn^+2^	1	CO_3_^−2^	1	H_2_O				
ZnSO_4_:_1_H_2_O(s)	−5.898	−5.626	1	Zn^+2^	1	SO_4_^−2^	1	H_2_O

**Table 5:** Component and percent of total concentration

Component	% of total concentration	Species name	Component	% of total concentration	Species name
CO_3_^−2^	0.084	HCO_3−_		1.613	FeH_2_PO_4_^+2^
	99.913	H_2_CO_3_ (aq)		96.921	FeHPO_4+_
K^+1^	98.287	K_+1_		0.037	CaH_2_PO_4+_
	1.704	KSO_4−_	SO_4_^−2^	56.691	SO_4_^−2^
Na^+1^	98.329	Na_+1_		1.187	HSO_4−_
	0.01	NaCl (aq)		0.018	AlSO_4+_
	1.661	NaSO_4−_		2.318	ZnSO_4_ (aq)
Ca^+2^	75.365	Ca_+2_		0.273	Zn(SO_4_)_2_^−2^
	0.023	CaCl+		1.215	FeSO_4_ (aq)
	24.612	CaSO_4_ (aq)		22.449	FeSO_4+_
Mg^+2^	78.963	Mg_+2_		2.12	Fe(SO_4_)_2−_
	0.038	MgCl+		1.975	MnSO_4_ (aq)
	20.999	MgSO_4_ (aq)		1.881	MgSO_4_ (aq)
Al^+3^	16.406	Al_+3_		9.599	CaSO_4_ (aq)
	0.059	AlOH_+2_		0.023	SrSO_4_ (aq)
	74.492	AlSO_4+_		0.09	NaSO_4−_
	9.036	Al(SO_4_)_2−_		0.018	KSO_4−_
Mn^+2^	80.258	Mn_+2_		0.128	NH_4_SO_4−_
	0.011	MnCl+	H_4_SiO_4_	99.833	H_4_SiO_4_
	19.732	MnSO_4_ (aq)		0.167	H_4_SiO_4_SO_4_^−2^
Zn^+2^	74.832	Zn_+2_	NH_4_^+1^	96.752	NH_4_^+1^
	0.026	ZnCl+		3.248	NH_4_SO_4−_
	23.743	ZnSO_4_ (aq)	NO_3_^−1^	99.235	NO_3_^−1^
	1.4	Zn(SO_4_)_2_^−2^		0.11	ZnNO_3+_
Pb^+2^	54.562	Pb_+2_		0.07	MnNO_3+_
	0.22	PbCl+		0.565	CaNO_3+_
	43.636	PbSO_4_ (aq)		0.011	NaNO_3_ (aq)
	1.581	Pb(SO_4_)_2_^−2^	Cl^−1^	99.11	Cl^−1^
AsO_4_^−3^	6.763	H_3_AsO_4_		0.104	ZnCl+
	0.029	HAsO_4_^−2^		0.375	CaCl+
	93.207	H_2_AsO_4−_		0.182	FeCl^+2^
Cd^+2^	73.142	Cd_+2_		0.044	MnCl+
	0.881	CdCl+		0.012	FeCl+
	23.705	CdSO_4_ (aq)		0.143	MgCl+
	2.271	Cd(SO_4_)_2_^−2^		0.023	NaCl (aq)
Cu^+2^	75.92	Cu_+2_	Fe^+3^	5.518	Fe^+3^
	0.017	CuCl+		29.058	FeOH^+2^
	24.045	CuSO_4_ (aq)		5.231	Fe(OH)_2+_
	0.016	CuHSO_4+_		3.43	Fe_2_(OH)_2_^+4^
Sr^+2^	78.14	Sr_+2_		1.735	Fe_3_(OH)_4_^+5^
	0.014	SrCl+		0.01	FeCl^+2^
	21.846	SrSO_4_ (aq)		52.496	FeSO_4+_
PO_4_^−3^	1.273	H_2_PO_4−_		2.479	Fe(SO_4_)_2−_
	0.066	H_3_PO_4_		0.041	FeHPO_4+_
	0.088	FeH_2_PO_4+_	Fe^+2^	74.403	Fe^+2^
				25.591	FeSO_4_ (aq)

**Table 6:** Concentration and activity

Species	Concentration	Activity	Log activity	Species	Concentration	Activity	Log activity
Al(OH)_2+_	2.2817E−12	1.9421E−12	−11.712	HPO_4_^−2^	4.6841E−12	2.4585E−12	−11.609
Al(OH)_3_ (aq)	5.5105E−16	5.5497E−16	−15.256	HSO_4−_	0.00012503	0.00010642	−3.973
Al(OH)_4−_	5.6181E−19	4.7819E−19	−18.32	K^+1^	0.00011205	0.00009537	−4.021
Al(SO_4_)_2−_	2.3492E−07	1.9996E−07	−6.699	K_2_HPO_4_ (aq)	2.2222E−19	2.238E−19	−18.65
Al^+3^	4.2656E−07	1.0002E−07	−7	K_2_PO_4−_	3.019E−27	2.5696E−27	−26.59
Al_2_(OH)_2_^+4^	2.5468E−15	1.9328E−16	−15.714	KCl (aq)	1.0892E−08	1.0969E−08	−7.96
Al_2_(OH)_2_CO_3_^+2^	7.4845E−30	3.9284E−30	−29.406	KH_2_PO_4_ (aq)	2.7566E−12	2.7762E−12	−11.557
Al_2_PO_4_^+3^	6.3258E−16	1.4833E−16	−15.829	KHPO_4−_	1.5684E−15	1.3349E−15	−14.875
Al_3_(OH)_4_^+5^	7.8844E−22	1.403E−23	−22.853	KNO_3_ (aq)	1.9491E−11	1.963E−11	−10.707
AlCl^+2^	1.6503E−11	8.662E−12	−11.062	KOH (aq)	1.1367E−15	1.1447E−15	−14.941
AlH_3_SiO_4_^+2^	1.5198E−10	7.9769E−11	−10.098	KPO_4_^−2^	5.6987E−24	2.9911E−24	−23.524
AlHPO_4+_	9.3566E−12	7.964E−12	−11.099	KSO_4−_	0.000001942	0.000001653	−5.782
AlOH^+2^	1.5339E−09	8.051E−10	−9.094	Mg(NH_3_)_2_^+2^	2.6719E−23	1.4024E−23	−22.853
AlSO_4+_	1.9368E−06	1.6485E−06	−5.783	Mg^+2^	0.00074463	0.00039083	−3.408
AsO_4_^−3^	1.5138E−16	3.5497E−17	−16.45	Mg_2_CO_3_^+2^	5.4696E−30	2.8708E−30	−29.542
Ca(NH_3_)_2_^+2^	3.6995E−22	1.9417E−22	−21.712	MgCl+	3.5997E−07	3.064E−07	−6.514
Ca(NO_3_)_2_	3.2754E−21	3.2987E−21	−20.482	MgCO_3_ (aq)	1.2674E−27	1.2764E−27	−26.894
Ca^+2^	0.0030953	0.0016246	−2.789	MgHCO_3+_	3.1256E−22	2.6604E−22	−21.575
CaCl+	9.4413E−07	8.036E−07	−6.095	MgHPO_4_ (aq)	4.7849E−13	4.819E−13	−12.317
CaCO_3_ (aq)	1.0784E−26	1.086E−26	−25.964	MgOH+	1.0274E−12	8.7446E−13	−12.058
CaH_2_PO_4+_	6.9924E−10	5.9517E−10	−9.225	MgPO_4−_	2.5075E−20	2.1343E−20	−19.671
CaHCO_3+_	1.3162E−21	1.1203E−21	−20.951	MgSO_4_ (aq)	0.00019802	0.00019943	−3.7
CaHPO_4_ (aq)	1.4409E−12	1.4512E−12	−11.838	Mn(NH_3_)_2_^+2^	3.719E−22	1.952E−22	−21.71
CaNH_3_^+2^	1.9249E−12	1.0103E−12	−11.996	Mn(NH_3_)_3_^+2^	8.0717E−32	4.2365E−32	−31.373
CaNO_3+_	1.6959E−09	1.4435E−09	−8.841	Mn(NH_3_)_4_^+2^	9.1238E−42	4.7888E−42	−41.32
CaOH+	2.2442E−13	1.9102E−13	−12.719	Mn(NO_3_)_2_ (aq)	1.1632E−16	1.1715E−16	−15.931
CaPO_4_−	6.6682E−18	5.6757E−18	−17.246	Mn(OH)_4_^−2^	6.9077E−39	3.6256E−39	−38.441
CaSO_4_ (aq)	0.0010108	0.001018	−2.992	Mn^+2^	0.00084592	0.000444	−3.353
Cd(CO_3_)_2_^−2^	1.1596E−52	6.0862E−53	−52.216	Mn_2_(OH)_3+_	2.3645E−21	2.0126E−21	−20.696
Cd(NH_3_)_2_^+2^	3.6635E−22	1.9229E−22	−21.716	Mn_2_OH^+3^	4.2428E−14	9.9485E−15	−14.002
Cd(NH_3_)_3_^+2^	1.4354E−30	7.5338E−31	−30.123	MnCl+	1.1089E−07	9.4386E−08	−7.025
Cd(NH_3_)_4_^+2^	1.6565E−39	8.6945E−40	−39.061	MnCl_2_ (aq)	3.5429E−11	3.5681E−11	−10.448
Cd(NO_3_)_2_ (aq)	1.5517E−20	1.5627E−20	−19.806	MnCl_3−_	2.4545E−15	2.0892E−15	−14.68
Cd(OH)_2_ (aq)	3.0846E−21	3.1065E−21	−20.508	MnCO_3_ (aq)	1.0674E−25	1.075E−25	−24.969
Cd(OH)_3−_	7.1824E−31	6.1133E−31	−30.214	MnHCO_3+_	6.5837E−22	5.6038E−22	−21.252
Cd(OH)_4_^−2^	2.3891E−41	1.2539E−41	−40.902	MnHPO_4_ (aq)	2.1644E−12	2.1798E−12	−11.662
Cd(SO_4_)_2_^−2^	9.0828E−09	4.7672E−09	−8.322	MnNH_3_^+2^	9.3442E−13	4.9044E−13	−12.309
Cd^+2^	2.9257E−07	1.5356E−07	−6.814	MnNO_3+_	2.0949E−10	1.7831E−10	−9.749
Cd_2_OH^+3^	3.3483E−20	7.8512E−21	−20.105	MnOH+	8.3835E−12	7.1357E−12	−11.147
CdCl+	3.5252E−09	3.0005E−09	−8.523	MnSO_4_ (aq)	0.00020797	0.00020945	−3.679
CdCl_2_ (aq)	2.5121E−12	2.53E−12	−11.597	Na^+1^	0.00056343	0.00047957	−3.319
CdCO_3_ (aq)	1.7268E−29	1.7391E−29	−28.76	Na_2_HPO_4_ (aq)	3.7125E−18	3.7388E−18	−17.427
CdHCO_3+_	2.9533E−25	2.5137E−25	−24.6	Na_2_PO_4−_	1.632E−25	1.3891E−25	−24.857
CdHPO_4_ (aq)	1.4264E−15	1.4365E−15	−14.843	NaCl (aq)	5.9125E−08	5.9545E−08	−7.225
CdNH_3_^+2^	1.895E−14	9.946E−15	−14.002	NaCO_3−_	7.4809E−29	6.3674E−29	−28.196
CdNO_3+_	2.1605E−13	1.8389E−13	−12.735	NaH_2_PO_4_ (aq)	1.3862E−11	1.396E−11	−10.855
CdOH+	1.0089E−14	8.5877E−15	−14.066	NaHCO_3_ (aq)	2.1193E−23	2.1344E−23	−22.671
CdSO_4_ (aq)	9.4822E−08	9.5496E−08	−7.02	NaHPO_4−_	1.2215E−14	1.0397E−14	−13.983
Cl^−1^	0.00024976	0.00021258	−3.672	NaNO_3_ (aq)	3.4007E−11	3.4249E−11	−10.465
CO_3_^−2^	9.2043E−27	4.831E−27	−26.316	NaOH (aq)	3.8356E−15	3.8628E−15	−14.413
Cu(CO_3_)_2_^−2^	5.6164E−51	2.9478E−51	−50.53	NaPO_4_^−2^	3.2762E−23	1.7196E−23	−22.765
Cu(NH_3_)_2_^+2^	1.7593E−20	9.2342E−21	−20.035	NaSO_4−_	9.5175E−06	8.1009E−06	−5.091
Cu(NH_3_)_3_^+2^	2.366E−27	1.2419E−27	−26.906	NH_3_ (aq)	1.4195E−10	1.4296E−10	−9.845
Cu(NH_3_)_4_^+2^	6.7235E−35	3.5289E−35	−34.452	NH_4_^+1^	0.00040152	0.00034176	−3.466
Cu(NO_3_)_2_ (aq)	2.0228E−22	2.0372E−22	−21.691	NH_4_SO_4−_	0.00001348	0.000011474	−4.94
Cu(OH)_2_ (aq)	3.1248E−19	3.147E−19	−18.502	NO_3_^−1^	2.9771E−07	2.5339E−07	−6.596
Cu(OH)_3_−	1.7038E−25	1.4502E−25	−24.839	OH−	8.1149E−12	6.9071E−12	−11.161
Cu(OH)_4_^−2^	1.4733E−36	7.7328E−37	−36.112	Pb(CO_3_)_2_^−2^	2.7727E−50	1.4553E−50	−49.837
Cu^+2^	1.5184E−08	7.9696E−09	−8.099	Pb(NO_3_)_2_ (aq)	1.3009E−19	1.3101E−19	−18.883
Cu_2_(OH)_2_^+2^	3.7945E−21	1.9916E−21	−20.701	Pb(OH)_2_ (aq)	2.2793E−18	2.2955E−18	−17.639
Cu_2_OH^+3^	6.2868E−20	1.4741E−20	−19.831	Pb(OH)_3−_	5.4183E−26	4.6119E−26	−25.336
Cu_3_(OH)_4_^+2^	3.2625E−33	1.7123E−33	−32.766	Pb(SO_4_)_2_^−2^	3.952E−09	2.0743E−09	−8.683
CuCl+	3.3884E−12	2.8841E−12	−11.54	Pb^+2^	1.364E−07	7.1594E−08	−7.145
CuCl_2_ (aq)	8.4395E−17	8.4995E−17	−16.071	Pb_2_OH^+3^	1.7484E−17	4.0997E−18	−17.387
CuCl_3−_	1.5421E−22	1.3125E−22	−21.882	Pb_3_(OH)_4_^+2^	1.5516E−33	8.1438E−34	−33.089
CuCl_4_^−2^_1_	4.2762E−28	2.2444E−28	−27.649	Pb_4_(OH)_4_^+4^	1.3902E−36	1.055E−37	−36.977
CuCO_3_ (aq)	2.2511E−28	2.2671E−28	−27.645	PbCl+	5.4968E−10	4.6787E−10	−9.33
CuHCO_3+_	3.0511E−26	2.597E−26	−25.586	PbCl_2_ (aq)	2.0284E−13	2.0428E−13	−12.69
CuHPO_4_ (aq)	1.9472E−16	1.961E−16	−15.708	PbCl_3−_	4.0528E−17	3.4496E−17	−16.462
CuHSO_4+_	3.2167E−12	2.738E−12	−11.563	PbCl_4_^−2^	5.0348E−21	2.6426E−21	−20.578
CuNH_3_^+2^	3.3824E−14	1.7753E−14	−13.751	PbCO_3_ (aq)	1.1637E−27	1.172E−27	−26.931
CuNO_3+_	8.115E−15	6.9072E−15	−14.161	PbH_2_PO_4+_	3.8807E−14	3.3031E−14	−13.481
CuOH+	2.9984E−13	2.5521E−13	−12.593	PbHCO_3+_	3.4586E−24	2.9438E−24	−23.531
CuSO_4_ (aq)	4.8091E−09	4.8433E−09	−8.315	PbHPO_4_ (aq)	1.6514E−16	1.6631E−16	−15.779
Fe(NH_3_)_2_^+2^	1.8136E−21	9.5187E−22	−21.021	PbNO_3+_	3.2755E−13	2.788E−13	−12.555
Fe(NH_3_)_3_^+2^	8.2541E−31	4.3323E−31	−30.363	PbOH+	2.6917E−12	2.2911E−12	−11.64
Fe(NH_3_)_4_^+2^	1.6025E−40	8.4111E−41	−40.075	PbSO_4_ (aq)	1.0909E−07	1.0987E−07	−6.959
Fe(OH)_2_ (aq)	2.5099E−19	2.5277E−19	−18.597	PO_4_^−3^	6.6212E−21	1.5525E−21	−20.809
Fe(OH)_2+_	0.00023557	0.00020051	−3.698	SO_4_^−2^	0.0059696	0.0031332	−2.504
Fe(OH)_3−_	1.6563E−26	1.4098E−26	−25.851	Sr^+2^	8.7518E−06	4.5935E−06	−5.338
Fe(OH)_3_ (aq)	1.0881E−10	1.0958E−10	−9.96	SrCl+	1.5247E−09	1.2978E−09	−8.887
Fe(OH)_4−_	1.1199E−15	9.532E−16	−15.021	SrCO_3_ (aq)	9.5196E−30	9.5873E−30	−29.018
Fe(SO_4_)_2−_	0.00011164	0.000095023	−4.022	SrH_2_PO_4+_	5.2866E−13	4.4997E−13	−12.347
Fe^+2^	0.00037202	0.00019526	−3.709	SrHCO_3+_	3.7047E−24	3.1533E−24	−23.501
Fe^+3^	0.00024847	0.00005826	−4.235	SrHPO_4_ (aq)	1.9503E−15	1.9642E−15	−14.707
Fe_2_(OH)_2_^+4^	0.000077229	5.8609E−06	−5.232	SrNH_3_^+2^	3.4341E−15	1.8025E−15	−14.744
Fe_3_(OH)_4_^+5^	0.000026047	4.635E−07	−6.334	SrNO_3+_	6.592E−12	5.6109E−12	−11.251
FeCl+	3.077E−08	2.619E−08	−7.582	SrOH+	2.2381E−16	1.905E−16	−15.72
FeCl^+2^	4.5894E−07	2.4088E−07	−6.618	SrSO_4_ (aq)	2.4468E−06	2.4642E−06	−5.608
FeH_2_PO_4_+	1.6774E−09	1.4277E−09	−8.845	Zn(CO_3_)_2_^−2^	3.5822E−49	1.8802E−49	−48.726
FeH_2_PO_4_^+2^	3.0645E−08	1.6085E−08	−7.794	Zn(NH_3_)_2_^+2^	7.7717E−19	4.0791E−19	−18.389
FeHCO_3+_	1.4915E−22	1.2695E−22	−21.896	Zn(NH_3_)_3_^+2^	3.5763E−26	1.8771E−26	−25.727
FeHPO_4_ (aq)	1.4242E−12	1.4343E−12	−11.843	Zn(NH_3_)_4_^+2^	8.2616E−34	4.3362E−34	−33.363
FeHPO_4+_	1.8415E−06	1.5674E−06	−5.805	Zn(NO_3_)_2_ (aq)	1.2902E−17	1.2993E−17	−16.886
FeNH_3_^+2^	1.5771E−12	8.2778E−13	−12.082	Zn(OH)_2_ (aq)	2.0373E−14	2.0518E−14	−13.688
FeOH+	6.308E−11	5.3691E−11	−10.27	Zn(OH)_3−_	1.5315E−22	1.3036E−22	−21.885
FeOH^+2^	0.0013085	0.00068679	−3.163	Zn(OH)_4_^−2^	7.9083E−32	4.1508E−32	−31.382
FeSO_4_ (aq)	0.00012795	0.00012886	−3.89	Zn(SO_4_)_2_^−2^	0.00001439	7.5529E−06	−5.122
FeSO_4+_	0.0023639	0.0020121	−2.696	Zn^+2^	0.00076928	0.00040377	−3.394
H^+1^	0.00058883	0.00050119	−3.3	Zn_2_OH^+3^	4.1208E−13	9.6623E−14	−13.015
H_2_AsO_4−_	0.000096936	0.000082508	−4.084	ZnCl+	2.6229E−07	2.2325E−07	−6.651
H_2_CO_3_* (aq)	9.9913E−17	1.0062E−16	−15.997	ZnCl_2_ (aq)	2.5842E−11	2.6026E−11	−10.585
H_2_PO_4−_	2.4187E−08	2.0587E−08	−7.686	ZnCl_3−_	6.7044E−15	5.7066E−15	−14.244
H_2_SiO_4_^−2^	1.9947E−21	1.047E−21	−20.98	ZnCl_4_^−2^	1.0348E−18	5.4311E−19	−18.265
H_3_AsO_4_	0.000007034	0.000007084	−5.15	ZnCO_3_ (aq)	1.1145E−25	1.1224E−25	−24.95
H_3_PO_4_	1.248E−09	1.2569E−09	−8.901	ZnHCO_3+_	7.7473E−22	6.5942E−22	−21.181
H_3_SiO_4−_	5.5466E−11	4.721E−11	−10.326	ZnHPO_4_ (aq)	1.5279E−12	1.5388E−12	−11.813
H_4_SiO_4_	0.00034043	0.00034285	−3.465	ZnNH_3_^+2^	2.192E−11	1.1505E−11	−10.939
H_4_SiO_4_SO_4_^−2^	5.6811E−07	2.9818E−07	−6.526	ZnNO_3+_	3.2971E−10	2.8064E−10	−9.552
HAsO_4_^−2^	3.0294E−08	1.59E−08	−7.799	ZnOH+	3.2765E−10	2.7888E−10	−9.555
HCO_3−_	8.3841E−20	7.1362E−20	−19.147	ZnSO_4_ (aq)	0.00024408	0.00024581	−3.609

In Table 3, results from the ammonium oxalate extraction (method 5) of P1 and P2 are presented, providing a measure for the amount and composition of amorphous material. The results show that Fe(III), As and S are largely present as amorphous material and together comprise about 40–50 % of the sample. Comparing the analyses for P1 and P2, it is clear that the latter contains a slightly smaller amorphous component. The X-ray diffraction analyses showed that among the crystalline minerals, quartz, mica and feldspar dominate. This is in line with the total chemical analyses, which combined with the results from the ammonium oxalate extractions evidenced that SiO_2_ (about 25 %) and elements such as Al_2_O_3_ (8–10 %), K_2_O (1.5 %), Na_2_O (0.5 %), MgO (0.85 %) and CaO (0.70 %) are present in the form of crystalline minerals. Reflections from secondary oxyhydroxy sulphate minerals (jarosite, schwertmannite or tooeleite) or iron(hyr)oxides (lepidocrocite, ferrihydrite, goethite) were not observed.

Under the microscope, the suspended material and basal sediment layer (P1 and P2) and the interstitial sediment (P3–P7) appear as mostly silt to fine sand-size light yellow to yellowish brown cryptocrystalline material, which is slightly translucent in fine particles and semi-translucent to isotic in larger aggregates. Particles are equidimensional to irregular and have no microscopically observable internal structure (see Fig. 5) nor features indicative of the presence of cellular or multicellular organisms and their organic structural remains (e.g. cell walls). Angular detrital mineral fragments (mostly quartz, feldspar and mica), also of fine silt to fine sand size, are common as single grains and as embedded fragments in the cryptocrystalline aggregates.

## Discussion

### Sediment

The chemical data on the composition of the amorphous material evidence that this material consists of ferric iron oxyhydroxy sulphate minerals that are amorphous to poorly crystalline. Jarosite can be excluded since it would show up in the X-ray analysis and be evident from the relatively high K content of the amorphous material, which is not the case. In this distinctly acidic environment and with such composition, the occurrence of ferrihydrite in more than very subordinate amounts is improbable (see, e.g. España 2007) and it would show up in a distinctly brown colour of the sediment, which is not the case at all. Therefore, the only relevant minerals are schwertmannite (Bigham et al. 1990, 1996; Yu et al. 1999; Schwertmann and Cornell 2000), the related tooeleite (Morin et al. 2003; Nishimura and Robins 2008) or a mixture of both. It should be emphasized that poorly crystalline schwertmannite and tooeleite cannot be readily identified through X-ray diffractometry, for which reason we describe this compound as schwertmannite-type material. Most of the total As (73 % as As_2_O_5_) also dissolved in the NH_4_Ox/Ox extract. Whether this As is bound in the structure (both tooeleite and schwertmannite) or sorbed as arsenate (schwertmannite) is not clear, but sorption is well known and considered to be most likely (Murad et al. 1994; Waychunas et al. 1995; Carlson et al. 2002; Fukushi et al. 2003).

The results from method 5 show that amorphous Al and Si is hardly present, virtually all Si and Al being present in the form of crystalline mineral fragments. The same holds for the base metals (K, Na, Mg and Ca). P is clearly present in very minor amounts. Thus, about half of the sediment (P1 and P2) consists of schwertmannite-type material with an appreciable arsenate component. The other half consists of a fine-grained mixture of primary quartz, mica and feldspar with minor amounts of accessory minerals (e.g. Ti minerals) and perhaps traces of sulphidic ores. Lastly, C and N contents are very low, emphasizing the mineral nature of the sediment and the truly very minor contribution of microorganisms to the sediment contained in the terrace rims.

Though sediments from the various layers in the moss section (samples P3–7) were not fully analysed (e.g. no XRF data), the data presented in Table 3 demonstrate that over time variations in composition are limited and rather invariable schwertmannite-type material formed an important component of the material, trapped as sediment by or precipitated on the moss. The same holds for As fixation/sorption.

### Moss and Its Coatings

Quite informative are the data in Table 2. Excluding those elements that are evidently contained in the living plant material (e.g. K, Mg and P), two types of coatings can be distinguished: thin translucent early coatings, largely composed of Fe and Al hydroxides with minor amounts of As and very low S contents, and later dense creamy coatings, which in addition to these hydroxides contain S and As. Moreover, in this subsequent stage, detrital material is incorporated and the composition is closer to that of the amorphous component of the sediment. It should be emphasized that these observations concern the ‘early precipitates’ since all green moss samples were thoroughly washed to remove fine interstitial material that did not adhere to this moss. Results for the latter material (P1–P2) evidence that this material is much lower in Al and contains less S, whereas the fixation/sorption of As seemingly has continued to reach rather extreme values (molar ratios of As/(As + S) are between 0.18 and 0.22), which is not yet the maximum ratio for As sorption on schwertmannite to become unstable (Carlson et al. 2002; Fukushi et al. 2003).

At first sight, puzzling are the relatively high Al contents (Al/Fe molar ratios are about 1.2–1.6) of the early precipitates (see Table 2), since in the amorphous component of the sediment Al contents are much lower (see Table 3). We attribute this to preferential adsorption of the trivalent Al^3+^ ion (and Fe^3+^) by the living moss that has a high cation exchange capacity, implying that upon its senescence and death, such adsorption capacity disappears and precipitation of schwertmannite-type material and As fixation/sorption prevails.

### Stream Water Chemistry

In 2008, Loayza-Muro et al. (2010) sampled stream water at the same location over four seasons and in triplicate. Water samples were not filtered and for estimation of metals were acidified with 10 M HNO_3_ prior to their analysis by ICP-OES. Their results thus included metals that were present as acid-soluble fine suspended material and cannot be compared with our results. Moreover, S content was not established, for which reason a check on their data based on the electroneutrality principle is impossible (see also Section 4.2). For details on the methods used, reference is made to Loayza-Muro et al. (2010). Over the past years, the mines and ore treatment plants operated intermittently and discharge of acid mine drainage varied over the years. This may explain the significant differences between the 2008 data and our 2010 data, but we cannot exclude analytical errors, since pH and EC values are similar to our values, but electrolyte contents are distinctly lower, which is hard to explain.

Based on our 2010 data, ion and ion pair concentrations were calculated, and ion activity products were compared with equilibrium constants of various relevant minerals (data not presented). Saturation indices indicate that potentially schwertmannite, tooeleite (both might contain arsenic) and jarosite might precipitate. Jarosite can be detected by X-ray analysis, but as already stated was not observed. This leaves schwertmannite/tooeleite (the ‘schwertmannite-type’ material) as the main precipitate to be formed in the stream on the basis of our water data, in conformance with our actual observations.

We only sampled once at the end of the dry season, and thus, our chemical stream water data are by their own no reliable estimate for the longer-term stream water composition. Much more decisive information on this composition can be deduced from our data on the chemical composition of the sediment captured in the moss rim (samples P3–7, Table 3) and the layered structure of this rim.

Seasonal variations in growth have been reported for many bryophytes, including *Bryum* species (e.g. Schwarz et al. 1992; Núñez-Olivera et al. 2010) and many aquatic or semi-aquatic mosses (e.g. Ilyashuk 2002; Guo et al. 2013). Assuming that the observed layering is indicative for seasonal variations in growth and environmental conditions (dry and wet season), a rough estimate of the growth rate of the moss rims can be obtained, being in the order of 4–5 mm/year (see also Fig. 3). This is well in accordance with growth rates observed in similar extreme environments, such as for *Bryum pseudotriquetrum* in continental Antarctica (Selkirk and Skotnicki 2007) and for mosses in Arctic lakes and streams (e.g. Sand-Jensen et al. 1999). Moreover, accumulation rates in Bryophyte dominated mires and peats in the high-altitude Peruvian Puna peatlands were found to be in the order of 2 mm/year (Salvador et al. 2014/15). Given the number of layers encountered in the highest terrace rims (up to 30), the age of the terrace rims would be in the order of at least several decades, with a growth rate of around 5 mm/year, which seems a realistic estimate for this truly stressed semi-aquatic environment.

Taking into account that during the dry season concentrations of solutes will be highest and schwertmannite-type material may precipitate, we can conclude that at least over several decades dry season stream water composition met the conditions required for such precipitation. España et al. (2005) and España (2007) described these as oxygenated, a pH between 2 and 4 and very high Fe^3+^ concentrations in the form of ferric sulphate complexes. This is clearly in line with our observed stream water composition.

### The Moss Microterraces

Terrace growth forms are not uncommon for bryophyte species, such as *Nardia compressa* and *Scapania paludosa*. In Alaska, these species formed 30–50-cm-wide terraces in a small stream, which impounded the swiftly flowing rivulet in a series of small pools (Shacklette 1965). This study by Shacklette (1965), however, remains one of the few studies describing terrace forming mosses from acid aquatic environments, whereas they are quite common in calcareous streams and springs: travertine terrace walls are often partly formed by bryophytes such as *Palustriella commutata* (e.g. Lang and Lucas 1970; Pentacost and Zhaohui 2002; Pentacost 2005).

España et al. (2007) reported ‘iron terraces’ from acid mine drainage systems, but their formation is ascribed to microbial activity and not to bryophytes. Evidently within our dense moss-built terrace rims, microbial activity may play a role since oxygen levels will be much lower than in the turbulent water. However, we did not find any indication for such activity to play a visible role in the formation of the terraces: the rims are dominantly composed of moss and its necromass, and the formation of the encrustations and coatings clearly is a secondary process, nor did we observe microstructures indicative for such microbial activity, as for example described by España et al. (2007).

It is not clear whether the terrace growth form is typical for *A. prostratum* since more detailed descriptions of its habitat do not exist. *A. prostratum* is an acrocarpic species without feathery branches, but it does have small leaves and a tufted growth form, which may increase encrustation. In our case, the encrustations are definitely not by calcite nor can photosynthesis be expected to play a serious role in their formation. Encrustation is rather a consequence of the successive formation of precipitates from supersaturated stream water, combined with ‘capture’ of fine detrital minerals.

Heavy metals such as Fe and also Al, and metalloids such as As, are known to be toxic to plants. Fe toxicity is mainly a problem under reduced conditions, because the oxidized form Fe(III) is much less soluble than the reduced Fe(II) form. In the terrace walls, Fe is relatively harmless due to its precipitation as schwertmannite-type material. Al is always toxic if present in large quantities, but concentrations in the stream water are low. Moreover, the precipitation of Fe and Al hydroxides in the early phase described above and prominent ion pair formation (data not shown) must lead to very reduced concentrations of dissolved Al^3+^ and Fe^3+^ near the living moss (see, e.g. España 2007). Arsenic may also be very toxic, partly because it is disrupting P-dependent aspects of metabolism (Finnegan and Chen 2012), but toxic levels in the vicinity of the living moss parts are most probably inhibited by the sorption of As to the schwertmannite-type precipitate.

## Conclusions

In the harsh aquatic environment of the Rio Santiago streambed, on living outer parts of the single higher plant species, the rare moss *A. prostratum* (Müll. Hal.) Besch, a coating of Al and Fe hydroxides is formed, which can be attributed to local supersaturation as a result of prevalent adsorption of trivalent cations by the living moss. Continued deposition ultimately induces its senescence and death, whereas newly formed leaves temporarily escape such fate. On the necromass and in the stream water, schwertmannite-type material precipitates to form moss necromass coatings and sediment particles that also contain ‘captured’ fine detrital primary minerals. Sorption or fixation of As by this material is prominent, leads to levels of up to 3.5 % As and most probably limits the concentration of dissolved arsenate. This is assumed to create a local, far less toxic environment around the living moss that allows this particular species—*A. prostratum*—to survive. The sequence of processes explains the peculiar terrace-forming growth of the moss, i.e. analogue to travertine-terrace formation in highly calcareous streams. Remarkably, this case study seems to be the first more detailed study on moss-built microterraces in a truly acid, aquatic environment.

Bryophytes have no roots and thus escape the encrusted and toxic parts of the terrace walls. Some species are more tolerant to heavy metals and As than others, especially calcifuge species (Bates 1978) or have developed particular tolerances to heavy metals (Shaw 198 7). Anyway, heavy metal concentrations in our moss are low and do not exhibit a clear relation with their concentrations in the stream water. Moreover, our study shows that estimates of these plant concentrations are problematic due to the presence of coatings and crusts and that results strongly depend on the pretreatment of the plant material. This calls for a critical evaluation of the bioindicator value of bryophytes in such polluted aquatic environment (see e.g. Samecka-Cymerman et al. 2002).

Solute concentrations and precipitation in the highly acid stream water are typical for acid mine drainage-polluted rivers (high in ferric iron, sulphate and calcium; schwertmannite-type precipitate) and have been so for a prolonged time, evidenced by the rather invariable composition of interstitial sediment from a moss-built terrace rim. Results furthermore show that the solute concentration of As is a poor indicator for the stream water quality, since it lies far below the levels encountered in fine sediment, which abounds in this water (as suspended material) and in the river deposits. Evidently, it would be hazardous to judge environmental risks of the use of such stream water by its solute composition and to overlook this fine, suspended load.

## Electronic supplementary material

ESM 1 (PDF 72 kb)

ESM 2 (JPEG 1680 kb)

ESM 3 (JPEG 1714 kb)

ESM 4 (JPEG 1650 kb)

ESM 5 (JPEG 1863 kb)

ESM 6 (JPEG 2782 kb)

ESM 7 (JPEG 1788 kb)

ESM 8 (JPEG 1567 kb)

ESM 9 (JPEG 1452 kb)

ESM 10 (JPEG 1695 kb)

ESM 11 (JPEG 1707 kb)
