# Supplementary material for: Rare Moss-Built Microterraces in a High-Altitude, Acid Mine Drainage-Polluted Stream (Cordillera Negra, Peru)
Source: Water Air Soil Pollut. 2015 May 29;226(6):201. doi: 10.1007/s11270-015-2390-x (PMC4446526; doi:10.1007/s11270-015-2390-x)
Supplement: Supplementary file 1 — (PDF 72 kb) [file 11270_2015_2390_MOESM1_ESM.pdf]

Supplementary material

1. Pictures of the block cut from one of the terrace rims and sampled for detailed analyses. Location of the block cut is visible in fig. 14.

Fig 6: Section of block (for location of sampling, see fig. 14).
Fig 7: Section of block with layered structure.
Fig 8: Section of block with layered structure

2. Pictures showing details of the stream and moss terraces.

Fig. 9: Riverbed slightly higher upstream with hydrophobic moss surface.
Fig. 10: Detail of moss rim.
Fig. 11: Double terrace wall.
Fig. 12: Capture of fine sediment by moss rim.
Fig. 13: Details of capture of fine sediment.
Fig. 14: Cut through layered terrace wall with creamy sediment and thin top layer of living moss.
Fig. 15: Small terrace rim in riverbed.

3. Speciation and saturation indices for relevant minerals.

The output of the VISUAL MINTEQ program is presented in tables 4a-c.

The chemical analysis of the water sample did not include the estimation of the redox potential and thus provided no measured value for that parameter, nor for the ratio Fe^{2+}/Fe^{3+}. The approach was as follows:

- The stream water was turbulent and measured oxygen content was such (on average 5 mg/L) that the water was oxygen saturated and thus fully aerobic, given the atmospheric pressure at the altitude of sampling (about 60% of the pressure at sea level). Moreover, the dominant iron mineral species is schwertmannite, which is built up of ferric iron.
- Calculations were based on the assumption that 10% of the total dissolved iron was present as Fe^{2+}. This, considering the aerobic, oxygen saturated nature of the stream water, very probably is an overestimation of the concentration of Fe^{2+}, but was accepted as the potentially highest ratio for Fe^{2+}/Fe^{3+} that might occur in the stream concerned.
- Values presented concern the assumed above ratio, but calculations have also been carried out for Fe^{2+}/Fe^{3+} = 1:1 (not presented), with only slightly deviating results regarding saturation indices for the relevant minerals.
- The VISUAL MINTEQ version 3.0 program (KTH, Stockholm, Sweden) was used to calculate dissolved metal, sulphate, and arsenate speciation, and to estimate saturation indices for relevant minerals (e.g. Erten-Unal et al. 1998).
- Schwertmannite is not included in the VISUAL MINTEQ list of minerals for which saturation indices are estimated. However, values reported in recent studies are such that the solution must be saturated. Reference is made to e.g. Caraballo, M.A., Rimstidt, J.D., Macias, F., Miguel, J., Hochella M.F. (2013): Metastability, nanocrystallinity and pseudo-solid solution effects on the understanding of schwertmannite solubility. *Chemical Geology*, 360-361, 22-31.
