# Supplementary figures and images for: Rare Moss-Built Microterraces in a High-Altitude, Acid Mine Drainage-Polluted Stream (Cordillera Negra, Peru)
Source: Water Air Soil Pollut. 2015 May 29;226(6):201. doi: 10.1007/s11270-015-2390-x (PMC4446526; doi:10.1007/s11270-015-2390-x)

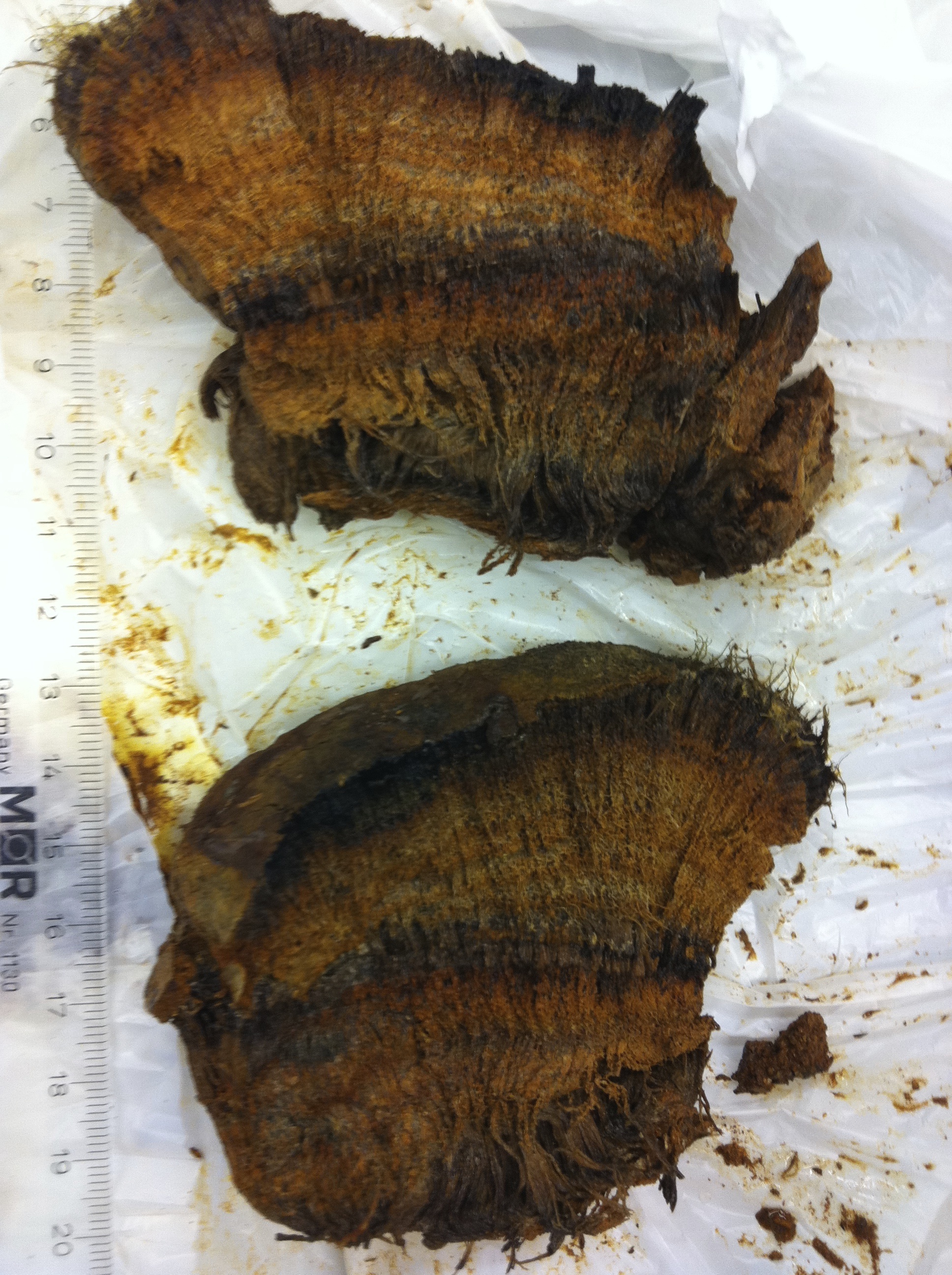

Supplement: Supplementary file 2 — (JPEG 1680 kb) [file 11270_2015_2390_MOESM2_ESM.jpg]

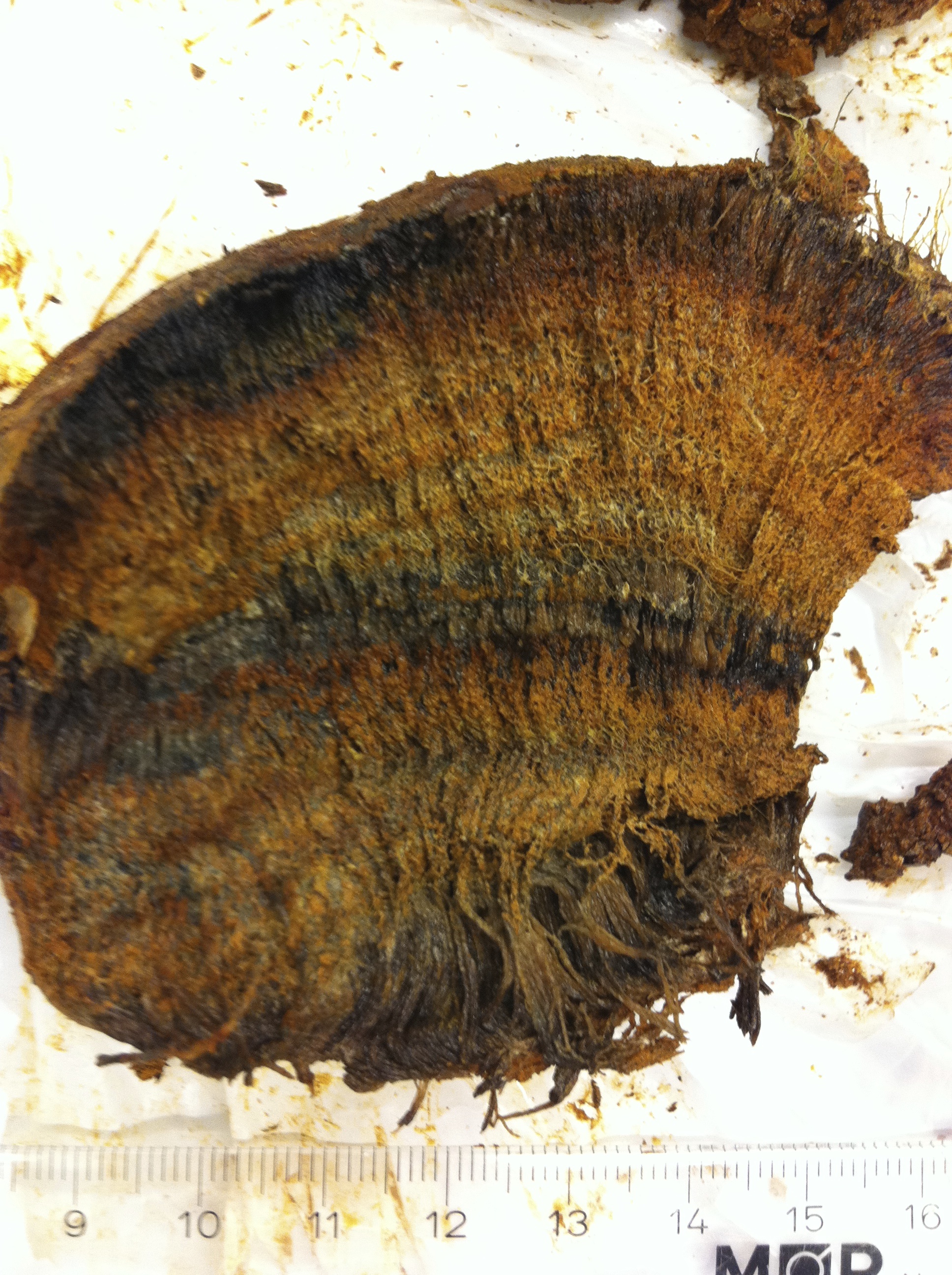

Supplement: Supplementary file 3 — (JPEG 1714 kb) [file 11270_2015_2390_MOESM3_ESM.jpg]

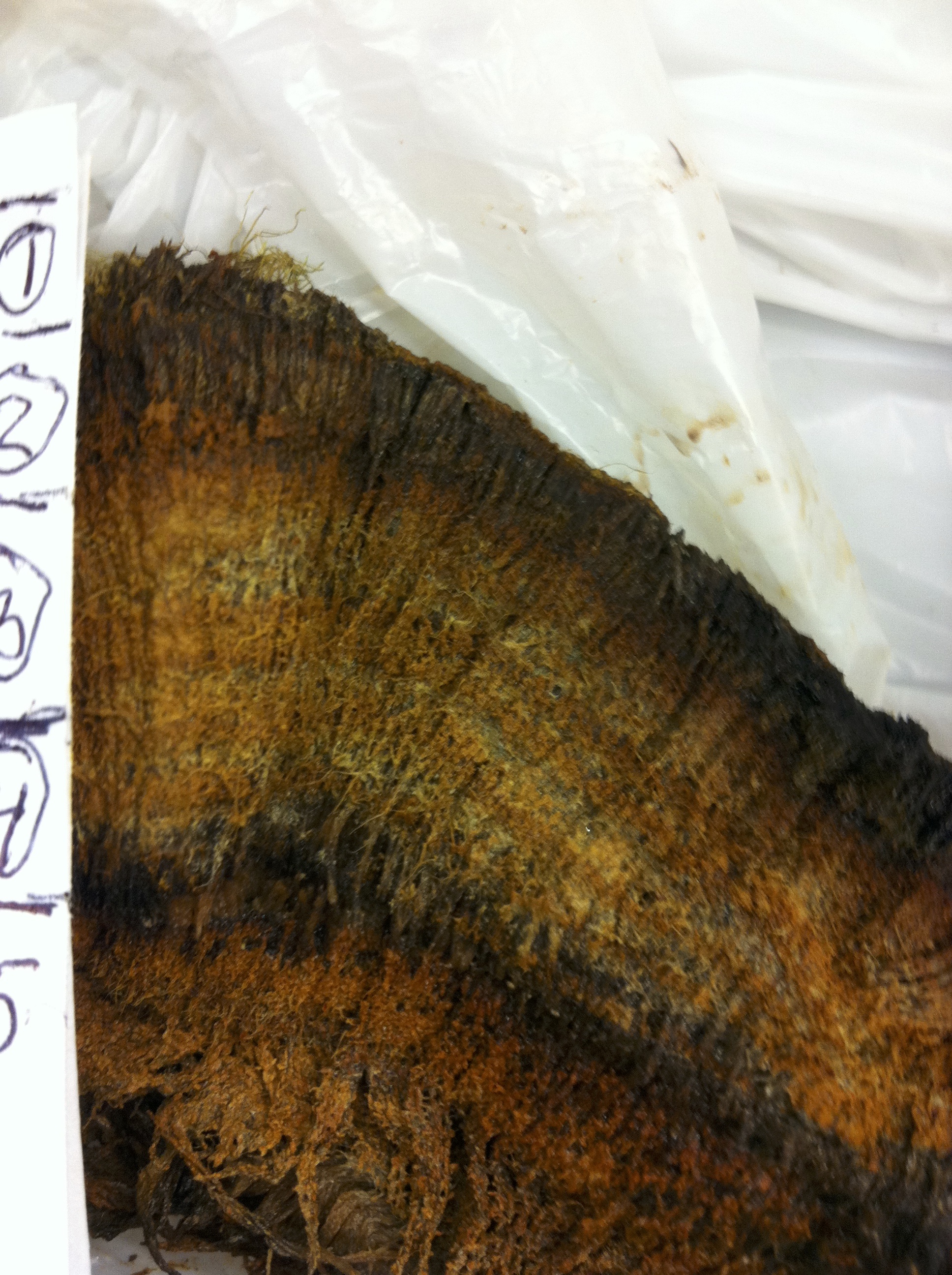

Supplement: Supplementary file 4 — (JPEG 1650 kb) [file 11270_2015_2390_MOESM4_ESM.jpg]

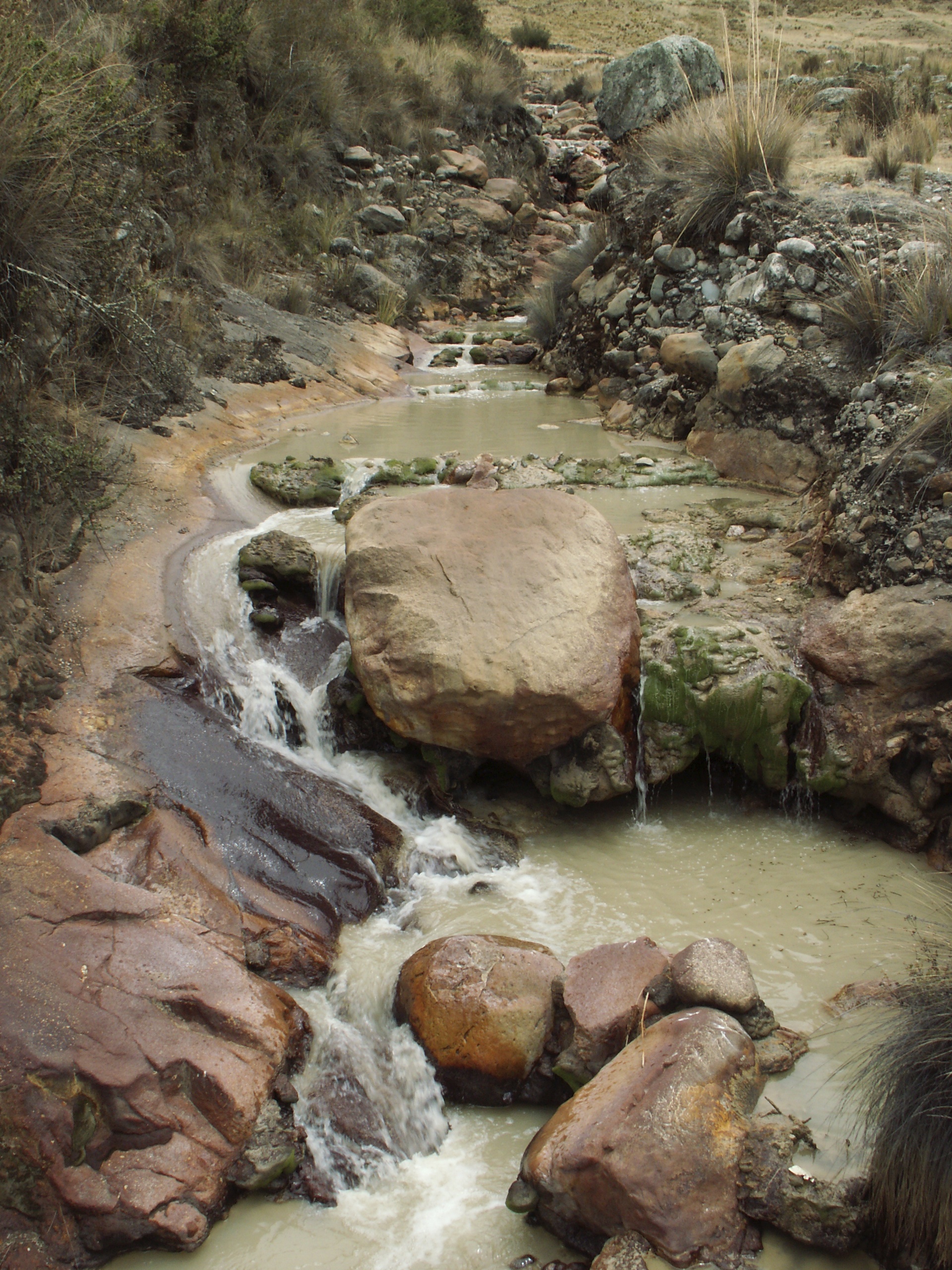

Supplement: Supplementary file 5 — (JPEG 1863 kb) [file 11270_2015_2390_MOESM5_ESM.jpg]

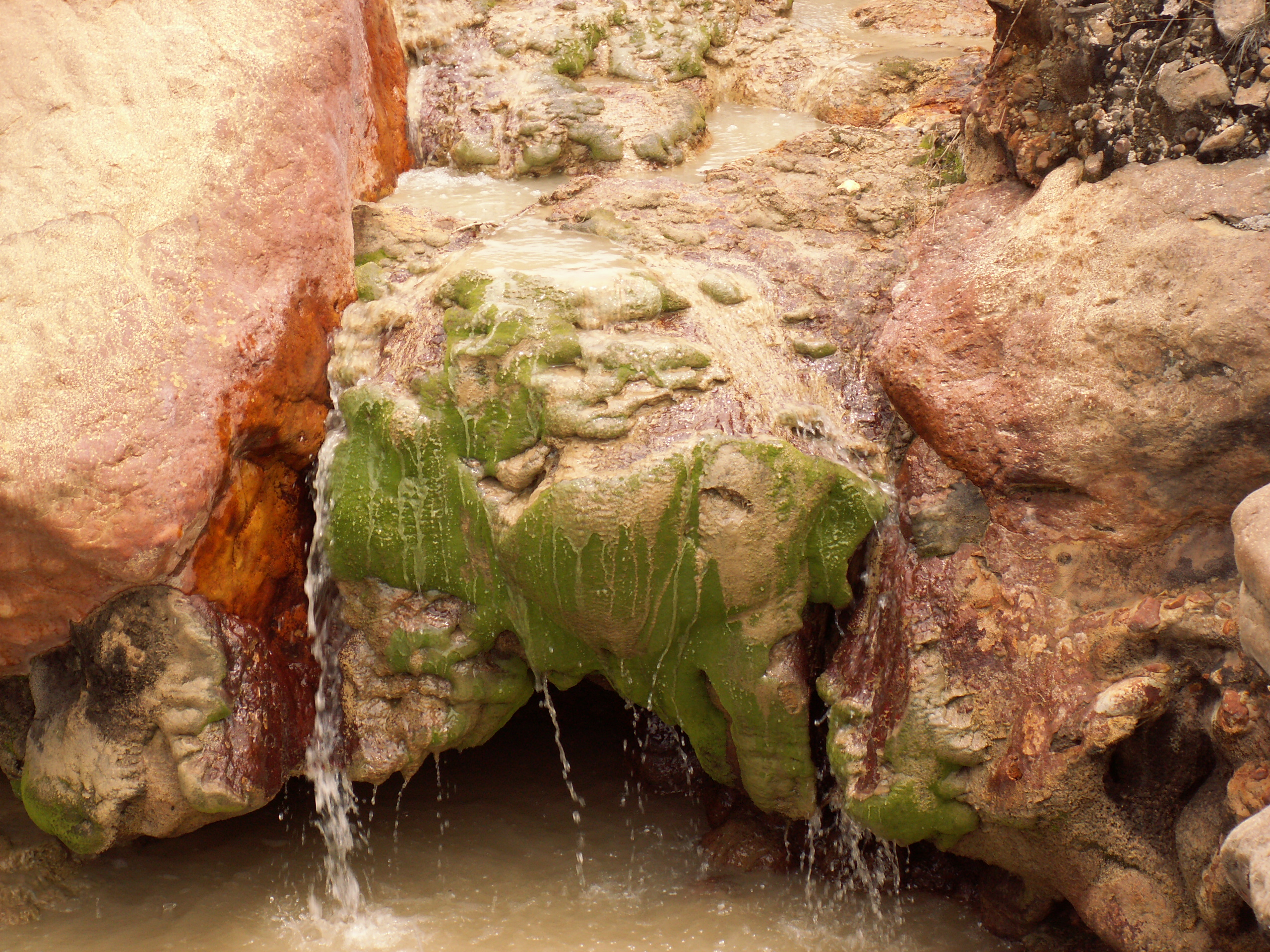

Supplement: Supplementary file 6 — (JPEG 2782 kb) [file 11270_2015_2390_MOESM6_ESM.jpg]

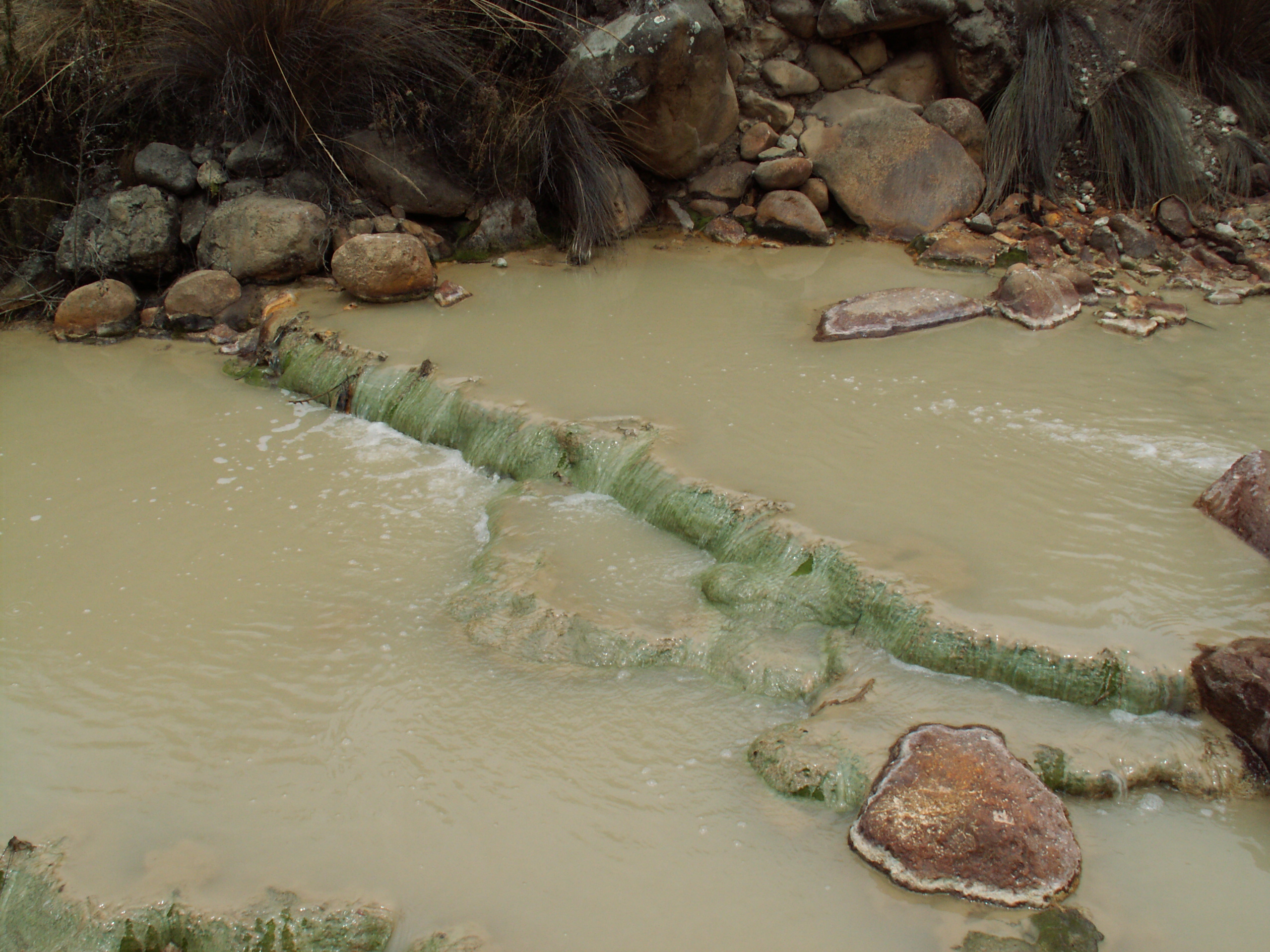

Supplement: Supplementary file 7 — (JPEG 1788 kb) [file 11270_2015_2390_MOESM7_ESM.jpg]

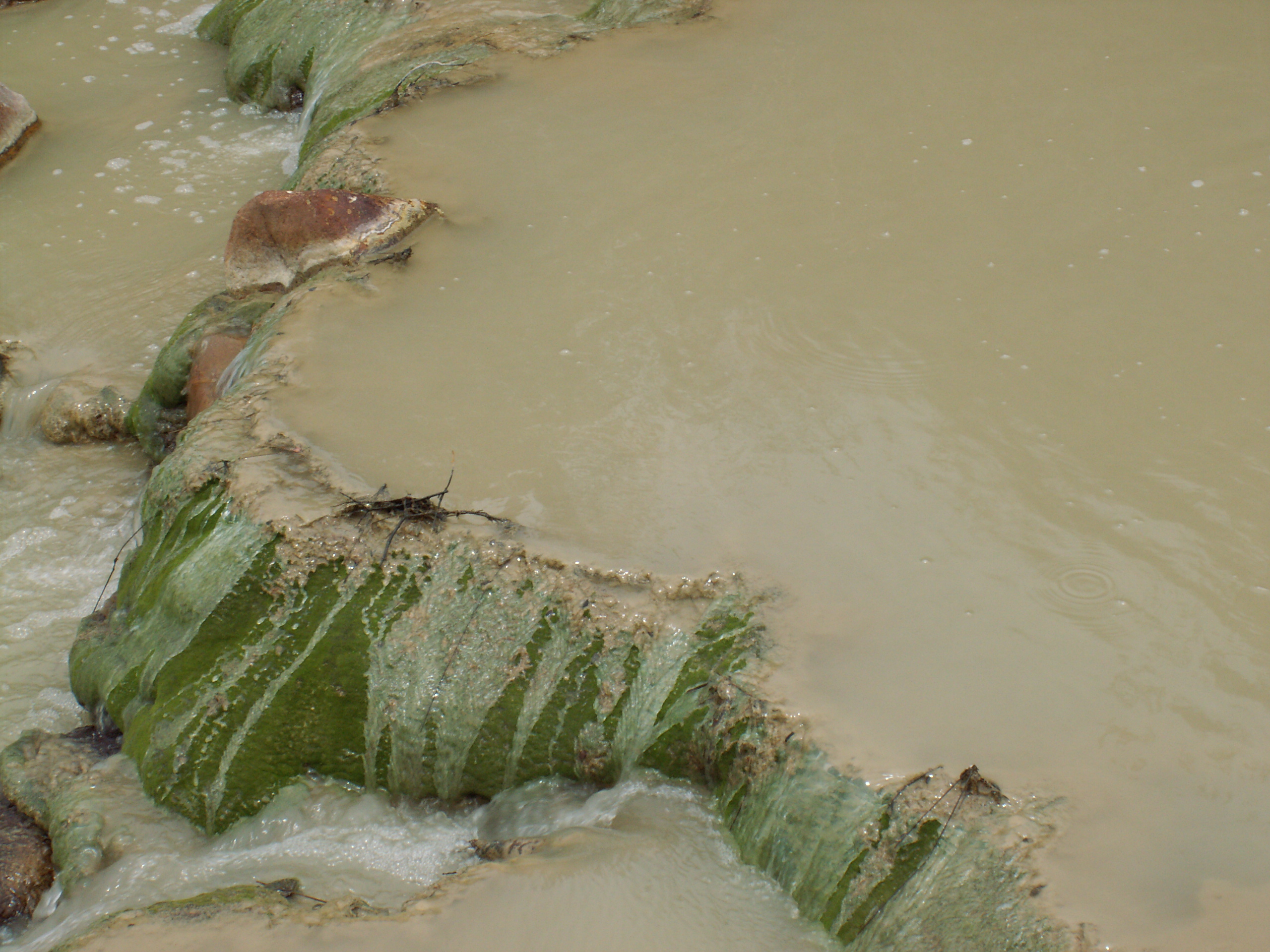

Supplement: Supplementary file 8 — (JPEG 1567 kb) [file 11270_2015_2390_MOESM8_ESM.jpg]

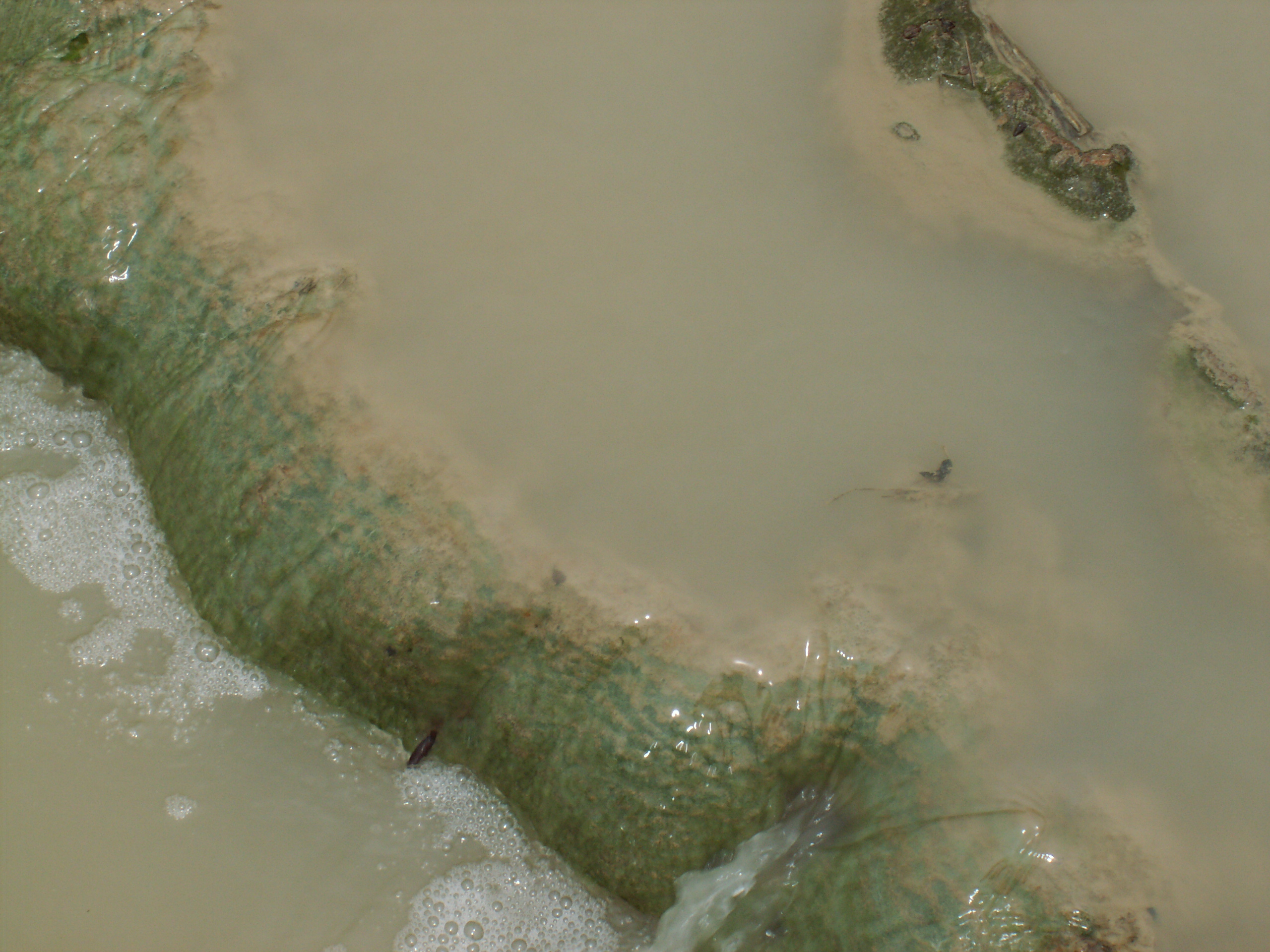

Supplement: Supplementary file 9 — (JPEG 1452 kb) [file 11270_2015_2390_MOESM9_ESM.jpg]

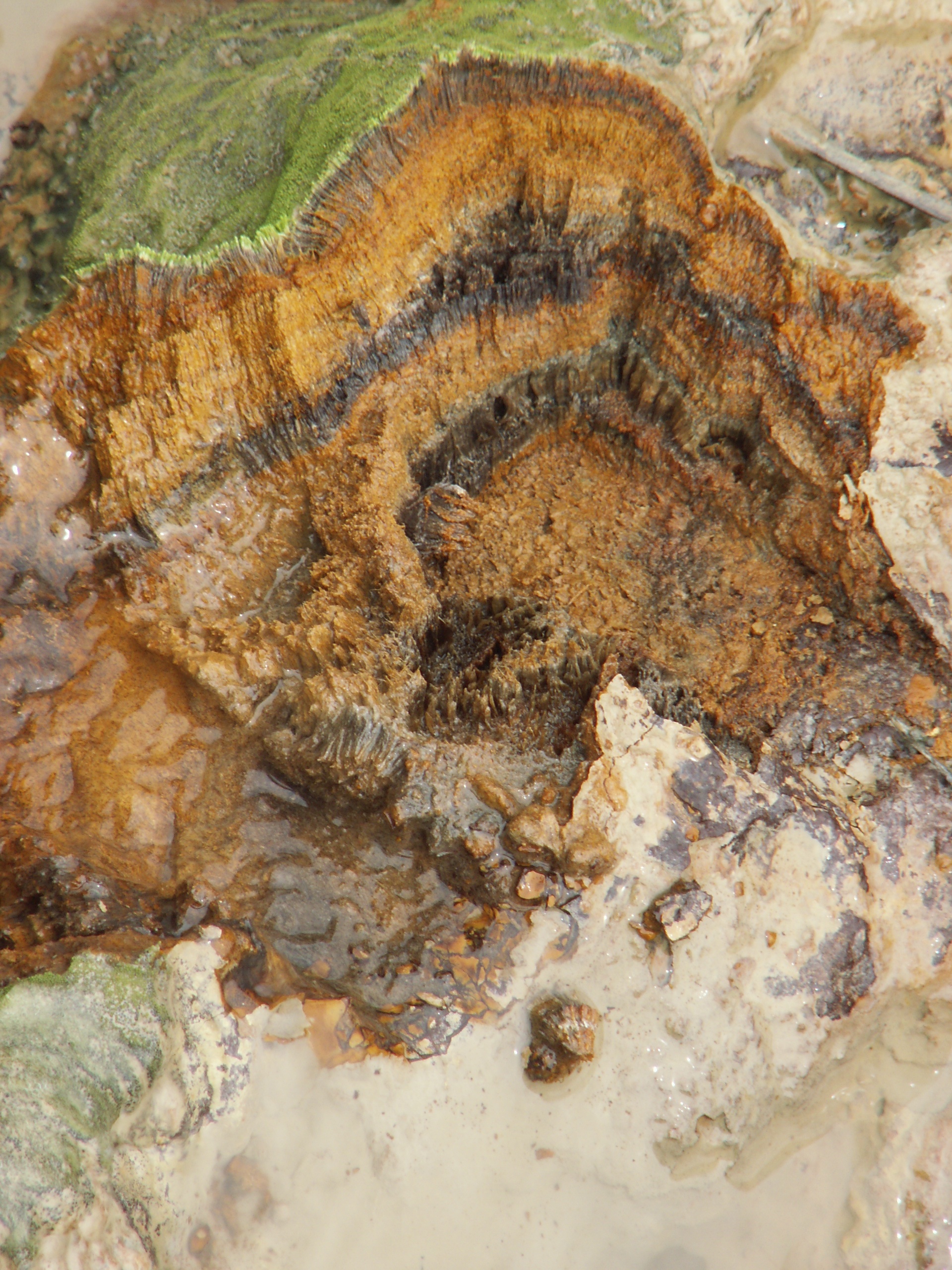

Supplement: Supplementary file 10 — (JPEG 1695 kb) [file 11270_2015_2390_MOESM10_ESM.jpg]

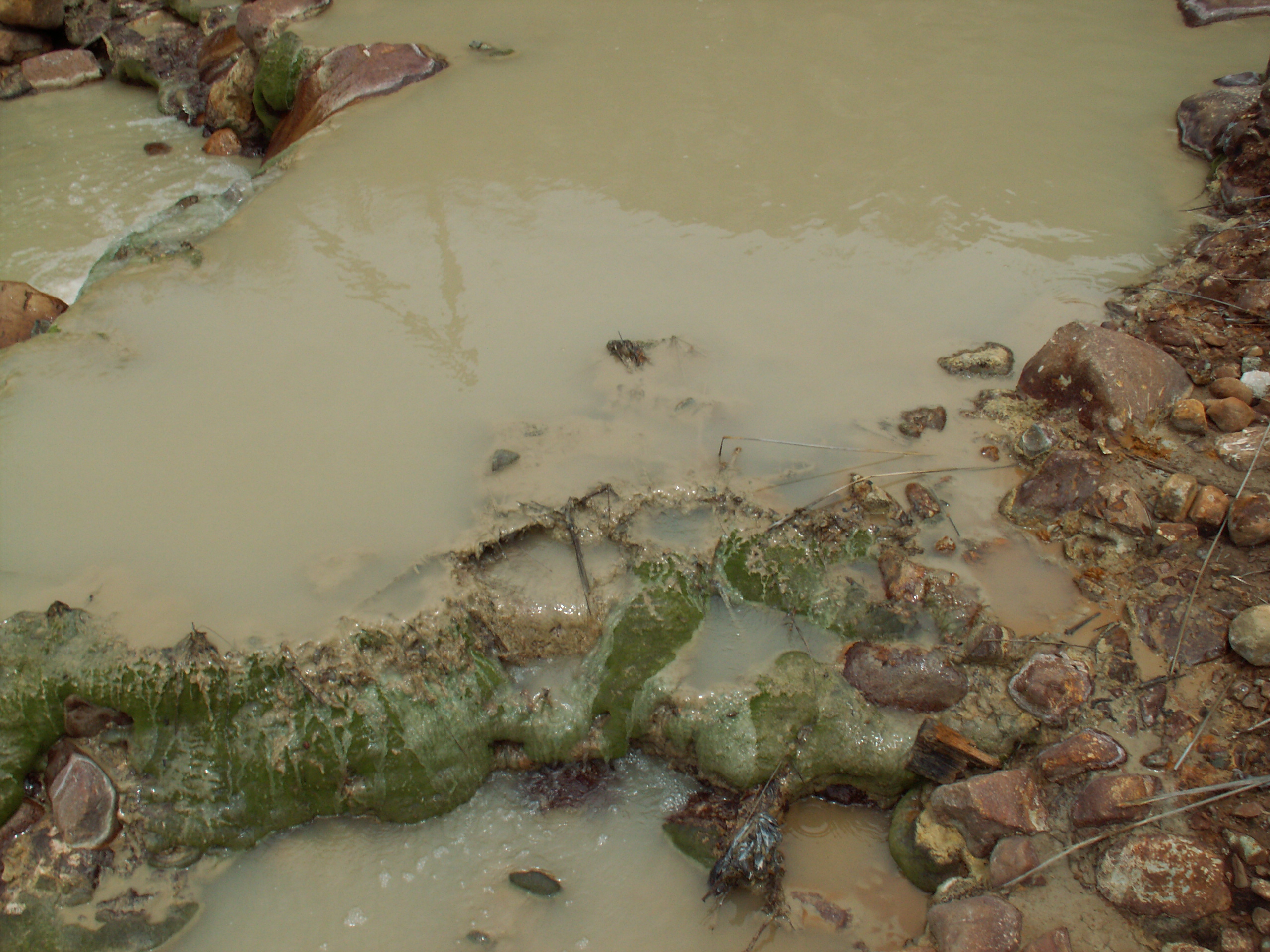

Supplement: Supplementary file 11 — (JPEG 1707 kb) [file 11270_2015_2390_MOESM11_ESM.jpg]
